# Therapeutic and Diagnostic Potential of Exosomes as Drug Delivery Systems in Brain Cancer

**DOI:** 10.3390/pharmaceutics15051439

**Published:** 2023-05-08

**Authors:** Dimitrios I. Avgoulas, Konstantinos S. Tasioulis, Rigini M. Papi, Anastasia A. Pantazaki

**Affiliations:** Laboratory of Biochemistry, Department of Chemistry, Aristotle University of Thessaloniki, 54124 Thessaloniki, Greece; avgoulas@chem.auth.gr (D.I.A.); ktasioul@chem.auth.gr (K.S.T.); rigini@chem.auth.gr (R.M.P.)

**Keywords:** exosomes, brain cancer, glioblastoma, drug delivery, natural nanocarriers, theranostics, treatment, diagnosis, personalized medicine

## Abstract

Cancer is designated as one of the principal causes of mortality universally. Among different types of cancer, brain cancer remains the most challenging one due to its aggressiveness, the ineffective permeation ability of drugs through the blood–brain barrier (BBB), and drug resistance. To overcome the aforementioned issues in fighting brain cancer, there is an imperative need for designing novel therapeutic approaches. Exosomes have been proposed as prospective “Trojan horse” nanocarriers of anticancer theranostics owing to their biocompatibility, increased stability, permeability, negligible immunogenicity, prolonged circulation time, and high loading capacity. This review provides a comprehensive discussion on the biological properties, physicochemical characteristics, isolation methods, biogenesis and internalization of exosomes, while it emphasizes their therapeutic and diagnostic potential as drug vehicle systems in brain cancer, highlighting recent advances in the research field. A comparison of the biological activity and therapeutic effectiveness of several exosome-encapsulated cargo including drugs and biomacromolecules underlines their great supremacy over the non-exosomal encapsulated cargo in the delivery, accumulation, and biological potency. Various studies on cell lines and animals give prominence to exosome-based nanoparticles (NPs) as a promising and alternative approach in the management of brain cancer.

## 1. Introduction

**Figure 1:**
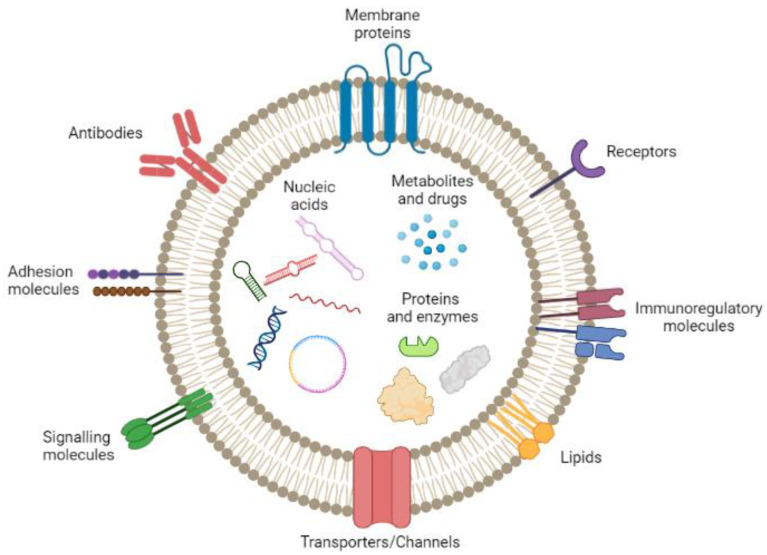
A representative scheme of exosomal composition. Created with Biorender.com.

Cancer accounts for millions of deaths globally [1]. Among all types, brain cancer remains very challenging due to its aggressiveness and poor treatment, resulting in low survival rates [2,3]. More specifically, glioblastoma (GBM) represents the most frequent and deadly brain malignant form, displaying a median survival rate of 8 months, which can reach up to 15 months if applying the existing treatment plans [4,5,6]. Currently, the main therapy strategies employed either alone or in combination, i.e., surgery, chemotherapy, radiotherapy, and immunotherapy exhibit challenges and side effects. For instance, sensitive brain tissue demands high surgical accuracy, while chemotherapy drug administration is linked to low specificity, high toxicity, poor efficacy, and drug resistance [1,7,8]. In addition to this, the major hurdle preventing drugs from reaching their target is the impermeability of the BBB, which is a structure that impedes brain access to substances owing to its complex physiology [8]. As a consequence, there is an imperative need for the generation of novel brain antitumor drug delivery systems in an attempt to simultaneously deal with cancerous cells and eliminate the deleterious effects of drugs on healthy tissues. In recent years, the contribution of nanotechnology in this field of research was tremendous, introducing nanoparticles (NPs) as a promising anticancer weapon for both therapeutic and diagnostic purposes [1,9,10]. However, synthetic drug-encapsulated NPs suffer from toxicity, immunogenicity, and low permeability through the BBB [9,11]. In order to overcome the limitations of synthetic NPs, researchers appealed to alternative solutions of natural NPs, such as exosomes. Exosomes constitute a class of cell-originated, phospholipid-bilayer vesicles with a diameter of 30–150 nm. These nanosized biovesicles are secreted by various cell types including macrophages, dendritic cells, natural killer cells, T cells, B cells, mesenchymal stem cells (MSCs), endothelial, epithelial cells, mast cells, adipocytes, platelets, fibroblasts, astrocytes, Schwann cells, neurons, and cancer cells [7,11,12]. They contain molecules such as deoxyribonucleic acid (DNA), ribonucleic acids (RNAs), proteins, lipids, and metabolites [13,14], thus functioning as natural delivery systems of biological agents throughout the organism. In addition to their encapsulated cargos, exosomes might also carry various biological agents on their membranes, such as adhesion, signaling, and immunoregulatory molecules, receptors, antibodies, lipids, proteins, transporters, and channels enabling them to interact with their cell targets [15,16,17]. A representative image of an exosome’s content is shown below (Figure 1). The biological composition depends on the cell source, the state of cell health, as well as the extracellular stimulus [18]. In terms of communication, cells can send and receive messages, even at long distances, utilizing the mechanism of exosome secretion by a donor cell and the uptake by a recipient. The transfer of cargo molecules that function as bio-mediators, inside the highly stable and protected environment provided by exosomes, affects crucial interactions and signal transduction between cells [3,7]. Biocompatibility, low immunogenicity, targeted delivery, convenient cell uptake, penetration through BBB, and the opportunity for a desired drug loading along with surface modifications are some properties that highlight the prospective brain anticancer activity of exosomes [19]. Moreover, they can be detected and isolated from various fluids including blood, urine, saliva, cerebrospinal fluid, ascites fluid, pleural effusion, amniotic fluid, bronchoalveolar fluid, breast milk, seminal plasma, tear fluid, and malignant ascitic fluid [20,21,22,23]. Although reviews regarding the employment of exosomes in brain cancer have been previously reported [2,5,7,24,25,26,27,28], this work aims to discuss comprehensively both the diagnostic and therapeutic potential of these nanocarriers covering the most recent literature on the topic. Physicochemical characteristics, biological properties, isolation methods, biogenesis and the internalization of exosomes are also summarized.

## 2. Physicochemical Characteristics of Exosomes

Nowadays, scientists provide a big arsenal of various natural nanosystems that utilize a spectrum of natural materials, including proteins, lipids, polysaccharides, synthetic polymers, and inorganic materials, differing in shapes, sizes, structures, and compositions [29,30,31,32,33]. Owing to the combination of advantageous physicochemical and biological characteristics of natural components, the aforementioned NPs have been recently assessed as auspicious fundamental materials for cancer therapy. In particular, natural-derived nanostructures possess desired properties, such as enhanced biocompatibility, biodegradability, non-immunogenicity, and superior safety profiles in contrast to the majority of synthetic NPs. Moreover, they own functional groups that promote their reaction with chemicals enabling their modification, thus leading to the achievement of more effective final formulations [30,32,34].

Additionally, due to the fundamental role of toxicology evaluations in the further clinical outcome assessment of particulate nano drugs [35], the propitious cellular response to natural NPs could their rapid exploitation in clinical tests, and subsequently, in various medicinal applications instead of synthetic alternative solutions. An extensive list of anticancer nanoparticle formulations containing resveratrol and curcumin (Cur) recording their physicochemical characteristics, the type of cancer, and crucial effects is reviewed in detail [36].

Despite the progress in this field, several concerns have been raised regarding the use of these NPs, relating to their bioavailability, expense, undesirable outcomes, including cytotoxic effects on diverse cells [37,38], biocompatibility, and a very limited half-life in vivo, which is revealed by the swift removal from the systemic circulation [39].

Exosomes are natural NPs able to transport and distribute a spectrum of proteins as well as some nucleic acids, mainly RNA, whereas they are also liable to subjection to the engineering process in order to ameliorate their effectiveness against tumor progression [40]. Interestingly, these nano-scale vesicles originated from membrane invaginations and originally participated in the communication between cells; they display penetration capacity through the BBB, which is an advantage that enables their utilization as vehicles for the transportation of administered therapeutic agents for brain diseases [24].

Exosome applications have emerged as an auspicious nanotechnology field expanded to several diseases, therefore contributing to both diagnosis and therapy. Owing to their innate properties, exosomes perform efficient drug transportation, exhibit high biocompatibility inside the organism, permeate easily through physiological barriers, and cause negligible or mild side effects, thus diminishing the distance until clinical tests and translational medicine [24]. A recent review highlights the attempts documented at drug transportation utilizing exosomes destined for neurodegenerative diseases and brain cancer therapy, while it also synopsizes the possible hindrances and future perspectives that will emerge in clinical translation [24].

Notably, exosomes regarding their properties excel beyond other NPs because of their remarkable biocompatibility, negligible immunogenicity, sufficient stability, protracted circulation half-life, recognition of their targets, and ability to cross physical barriers such as the BBB [41]. Additionally, their capability to carry active biomolecules with therapeutic and diagnostic potential as well as their intrinsic propensity for bioengineering has been greatly recognized nowadays.

### 2.1. Exosome Isolation

Taking the physical and chemical properties of extracellular vehicles (EVs) into consideration, exosome isolation from body fluids was attempted using several methods that aimed to ameliorate the yield, purity, structural integrity, and functionality of exosomes, leading indeed to a final preparation with altered parameters [42]. Based on the applied isolation method, the exosomal purity and the physicochemical characteristics could be affected [24]. The methods that have evolved to enhance the aforementioned exosomal parameters include several size-based techniques, such as tangential flow filtration (TFF) [43], ultrafiltration apparatus [44], size-exclusion chromatography (SEC) [45], polymer precipitation [46,47], ultracentrifugation [48], and microfluidics techniques [49,50].

An acoustic nano-filter could utilize ultrasound standing waves to apply electromagnetic wave forces on the biological liquid and permit the vesicles’ segregation depending on their mechanical properties such as their dimensions and density [51]. Currently, another standard protocol was reported involving a number of purification steps to achieve a suitable exosomal preparation [23], although the final batch-to-batch purity, as well as the sample concentration, is not sufficiently reproducible.

Concerning the employment of exosomes in medical applications and clinical use, a protocol that ensures the production of a sufficient amount of particles is required. Due to the high productivity of exosomal mass, which is necessary for clinical utility, TFF is regarded as the most convenient method, since it provides a higher yield with minor non-exosomal component contamination that usually occurs in serial centrifugations. In addition, high exosomal reproducibility has urged some corporations, involved in exosome commerce, to install TFF-based exosome manufacturing facilities [52].

According to a worldwide International Society for Extracellular Vesicles (ISEV) survey [53], ultracentrifugation (UC)-based exosome isolation is considered to be the most ordinarily utilized method [48,54,55,56,57,58,59]. Exosome isolation in gliomas has been described in detail [2]. By highlighting the lack of an established method to receive a homogeneous exosomal preparation, an exosome comparative quantification approach in terms of particles and protein concentration (μg/mL) was performed, depending on the isolation method, cellular origin, and source availability [2]. The benefits and drawbacks of exosomal purification methods have been recently reviewed [14,60].

### 2.2. Exosome Size

EVs differ in size depending on their type, ranging from 30 to 5000 nm, including exosomes, exomeres, microvesicles (MVs) or ectosomes, and apoptotic bodies, as well as in the mechanism of their biogenesis [61]. Even though the categorization of EVs, relying on exosomal size, is ceaselessly progressing [62], they are normally classified into four main subcategories containing

- Exosomes (30–150 nm);
- Microvesicles (100–1000 nm);
- Large oncosomes (1000–10,000 nm); and
- Apoptotic bodies (100–5000 nm) [63,64].

Other studies have reported that EVs are classified into three major subclasses including MVs or ectosomes, apoptotic bodies, and exosomes [65,66]. Their smaller size and unified shape permit exosomes to successfully elude the elimination by the mononuclear phagocyte system, leading to a prolonged circulation duration but also suggesting their supremacy concerning the communication between cells [61].

### 2.3. Exosome Stability

Regarding their stability, exosomes are proved to be adequately stable inside the majority of the body fluids, thus protecting the encapsulated biomolecules from catabolic processes. When the cells are under pathological conditions, they are subjected to alterations mirrored in the secretion of biological exosomal constituents. In the case of a differentiated exosomal cargo, this could be detected and correlated to the transcriptome, proteome, and lipidome analyses to gain insights. Consequently, the determination of fluctuations in the content of certain selected components will contribute to the exploitation of exosomes in the biomarker field [67].

Although exosomes represent a favorable cell-free therapy, a drawback regarding their storage stability exists, since they cannot be stored for an extended time period. Henceforth, to confront this problem, exosomes’ maintenance technology with the goal of keeping intact their biological potential and rendering them suitable for shuttle and clinical performance should be taken into consideration. Currently, the utilized shielding methods mostly comprise freezing, freeze-drying, and spray-drying [12]. Upon their preparation, when exosomes are lyophilized, they become more stable at higher temperatures [64].

Due to the distinct complexity of exosomes, their dimension range, and natural incompatibilities during their assembly, the inherent hazards that follow the biogenesis process are more than those of the synthetic production [68,69]. These properties reflect on the variability of their reproducible load with therapeutic drugs, which is something that requires suitable technological approaches [68].

### 2.4. Exosome Biological Properties

Exosomes own unique and inspiring properties that have motivated scientists to employ them for therapeutic approaches against many diseases, including degenerative disorders, cancers [61,62], and, mainly, brain cancer. Due to these suitable properties, their therapeutic benefits, and unelucidated functions, exosomes elicit the inquisitiveness of researchers to develop them as drug transportation systems [19].

Their advantageous properties are enumerated as the adequate stability in both physiological and pathological conditions, their small and relatively homogeneous dimensions, their ability for intracellular distribution of selected load through the fusion process of membranes, the capability to traverse physiological barriers, such as the BBB, the absence of immunogenicity and the autologous use permitting personalized medicine [33]. Another benefit that exosomes possess is attributed to their bio-originated membrane, which is inactive in the generation of the named “protein corona”, which is the abundant proteinaceous coating that usually cover NPs when they interact with proteins that are adsorbed to their surfaces in various biological processes [70,71].

On the contrary, among the disadvantages are the requirement of standardized protocols for their isolation and purification, the necessity of sufficient characterization of cell provenance, the undesirable outcomes attributed to the exosome constituents themselves, and the absence of standardized mass production protocols for clinical applications [33].

Because of the heterogeneity of small EVs and the existence of non-vesicular extracellular material, a debate has emerged concerning the composition and functional properties of exosomes. By utilizing high-resolution density gradient fractionation and direct immunoaffinity capture techniques, the constituents harbored by exosomes and other non-vesicle material, including nucleic acids and proteins, were precisely characterized [72].

Exosomes participate in a spectrum of functions because of the membrane-connected proteins, e.g., tetraspanins (CD63, CD81, CD9, CD37, CD53, and CD82), intercellular adhesion molecule-1 (ICAM-1), integrins, intracellular transportation and membrane fusion proteins, such as Ras-associated binding (Rab) and Annexins, proteins related to multi-vesicular bodies (MVBs) and the endocytic pathway, i.e., the endosomal sorting complex needed for transport (ESCRT). For instance, Alix, tumor susceptibility gene 101 (TSG101), proteins mainly participate in vesicle trafficking, such as cell surface receptors and antigen presentation molecules, such as major histocompatibility complex I and II (MHC-I, MHC-II), human leukocyte antigen-G (HLA-G) [73] and, in addition, other proteins implicated in long-distance signal transduction, such as cytokines [74], hormones [75], growth and transcription factors [76], and heat shock proteins (HSPs) [77,78]. Moreover, the protein content of the membrane depends on the cell type and the methodology of purification, as portrayed in the record of databases ExoCarta [54,79], Vesiclepedia [80] and EVpedia [81]. An abundant depository completes the exosomal composition, including cholesterol, sphingomyelin, phosphatidylcholine, phosphatidylserine, phosphatidylethanolamine, phosphatidylinositol, ganglioside GM3, prostaglandins, and lyso-bisphosphatidic acid [82,83].

There is evidence that exosomes comprise or express a variety of bioactive molecules including proteins, RNAs (mRNA, microRNA, and other non-coding RNAs), DNAs (mitochondrial DNA (mtDNA), double-stranded DNA (dsDNA), single-stranded DNA and viral DNA, lipids, amino acids, and metabolites. These miscellaneous constituents exert important roles, such as in intercellular signaling and regulating neighboring or remote cellular microenvironments [84,85,86]. Moreover, to obtain insights into exosomes composition, small EV exosomes, and non-vesicular components, a combination of the direct immune affinity capture method targeting the classical exosome marker tetraspanin with high-resolution density gradient fractionation was applied [14]. Although exosomes and non-vesicular components were found to bear different proteins and RNAs, they did not possess microRNA (miRNA) processing proteins, such as Argonautes (Agos), glycolytic enzymes, or cytoskeletal proteins. Moreover, others reported that exosomes did not contain double-stranded DNA (dsDNA) or DNA-binding histones, which are typically found in exosomes, suggesting that dsDNA or DNA-binding histones are released through an exosome-unmediated mechanism. These outcomes highlight the necessity for a revaluation of the exosome composition by an in-depth analysis to elucidate exosomal heterogeneity [14]. One idiosystatic and noteworthy property is that endogenous exosomes operate as “signalosomes” inasmuch as they transmit signals through ligands or adhesion molecules localized on the exosomal membrane or enclosed in the interior of the exosome. This communication carried out by the intervention of the exosome has several unique features [87]. Specifically, exosomes exchange various substances between the cells without contact and, as a result, participate in various ways in cell communication and signal transduction processes, thus comprising autocrine, endocrine, juxtracrine, paracrine, or in distance transmission [88].

The existence of exosomes in biofluids may exhibit certain diagnostic and therapeutic properties. For instance, the extensive investigation of circular RNAs (circRNAs) present in exosomes circulating in biological fluids, such as blood, or cerebrospinal fluid (CSF) may contribute to discovering the fundamental mechanisms of the progress of neuropsychiatric diseases [67]. Furthermore, miRNAs transported by serum exosomes are useful for the prognosis and diagnosis of spinal cord injury (SCI) [89]. The recognition of the therapeutic properties of exosomes renders them as candidates for utilization in clinical applications that are dependent on the three following features: the kind of vesicle and load and/or the type of surface modifications or functional encapsulated molecules [40].

### 2.5. Exosome Biogenesis and Internalization

According to the exosome biogenesis pathway, exosomes are derived from the endosomal membrane, creating a toward-the-inside budding, which leads to the generation of MVBs. Subsequently, MVBs possess two fates, either degradation into the lysosomes or fusion with the plasma membrane, and the subsequent externalization of exosomes.

**Figure 2:**
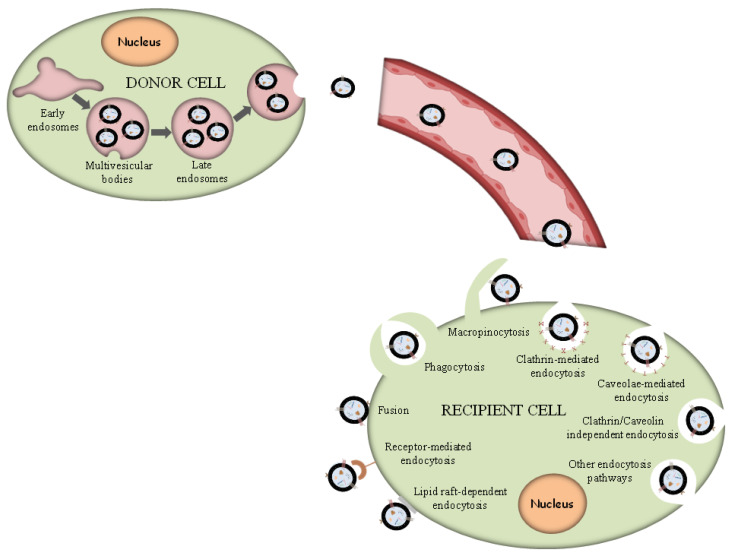
Exosome biogenesis and different uptake pathways.

Exosomes are supposed to constitute a homogeneous ensemble of vesicles that originate from the inner budding of the MVBs membrane. After MVBs fusion with the cellular membrane, the secretion of exosomes occurs, displaying the same membrane orientation as the parent cells [43]. For comparison, MVs constitute an almost heterogeneous group of vesicles shaped by exterior folding followed by disruption of the cell membrane, which could be controlled by some membrane lipid micro areas and some regulatory proteins, such as ADP Ribosylation Factor 6 (ARF6). Thus, both exosomes and MVs can be assumed as “signalosomes” implicated in diverse biological processes (Figure 2).

After their exocytosis, exosomes migrate into the extracellular matrix (ECM), where exosomal surface proteins assist in the detection of the target cells for the subsequent uptake [90]. Exosomes interact with particular receptors of the recipient cells by the intervention of integrins, tetraspanins and intercellular adhesion molecules, which subsequently internalize exosomes through the same conventional means following the uptake of various molecules into the cells, including:

(i) Phagocytosis. Phagocytosis is almost a similar internalization process to macropinocytosis where exosomes, along with some extracellular fluids, are phagocytosed. This process is common and adopted by both phagocytic cells, i.e., macrophages and dendritic cells (DCs) and non-phagocytic cells, i.e., T cells [91].
(ii) Macropinocytosis. Macropinocytosis is a process in which the deformation of the plasma membrane leads to the formation of protrusions that surround the extracellular fluid and exosomes, therefrom uptaking exosomes. This complicated process is mediated by many factors, since it relies on Ras-related C3 botulinum toxun substrate 1 (Rac1), actin and cholesterol and it requires Na^+^/H^+^ exchange [92].
(iii) Clathrin/caveolin-mediated endocytosis. The Clathrin protein shapes a basket knitted-like structure around the exosomes for their internalization. The plasma membrane of the target cells creates an internal folding, which is followed by squeezing off the clathrin-coated vesicle from the membrane. Subsequently, the exosome releases all its cargo in the targets cell’s endosomes to implement particular functions [93].
(iv) Internalization through lipid raft. The endocytosis process may be also mediated by caveolin-1, similar to the clathrin-dependent way, whose clusters in plasma membrane are configured in rafts. The invagination of the plasma membrane named caveolae is abundant in caveolin 1, glycolipids, and cholesterol [94], and
(v) Direct fusion with the plasma membrane. In this fusion process, the Lysosomal-Associated Membrane Protein 1 (LAMP-1) and glycoprotein type I transmembrane protein are implicated primarily residing across lysosomal membranes, and in certain cases, it can be expressed across the plasma membrane of the cell, and the integrins or tetraspanins [95,96].

The internalization of exosomes through the receptor-mediated endocytosis process has been reviewed extensively [92], and a number of receptor–ligand complexes listed are considered to facilitate exosomal internalization, while several other proteins have been identified as contributors in this endocytosis process of exosomes through protein–protein interactions implicated in the exosomal uptake. Apart from the detailed presented receptor/ligand complexes, other receptor-mediated endocytosis ligands and receptors have been also identified, but still, further evaluation is required to elucidate their role in exosomal internalization (Figure 2).

### 2.6. Cancer Cell-Derived Exosomes

Cancer cell-derived exosomes are pivotally involved in tumor-linked processes including proliferation, metastasis, and immune regulation [61,62], whereas they might be employed to surveil the progress of disease [63], serving as selected diagnostic markers. Although all cells possess the ability to generate exosomes, tumor-derived exosomes (TdEX) seem to display distinguishing characteristics from exosomes secreted by normal cells, being mightily immunosuppressive [97,98]. TdEX can transmit signals between cells or transport various biological active matter toward the recipient cells, in this way triggering alterations of their cellular features [97,98,99]. Another important property that should be taken into consideration is that exosomes and, especially, those originating from tumor cells are considered to preferentially perform delivery to tumors, which is a phenomenon attributed to their homotypic features. Due to these properties, the TdEX has received growing interest to be considered as a potential clinical biomarker, and they may constitute particular vehicles for specific cancer targeting. Moreover, the pharmacokinetic properties of naive exosomes and factors contributing their intracellular “luck or destiny” have been recently summarized [87].

Cancer cells-secreted exosomes convey pathophysiological features and function as intermediates of tumor progression and distant lesions genesis [70,71,72]. Several in vitro studies propose that in the micro-surrounding of a tumor, cancer cell-derived exosomes probably attack other neoplastic cells in preference to physiological cells, therefore employing them as selective distribution vehicles [100]. In addition, cancer cell-derived exosomes carry various DNA, including genomic, cytoplasmic, and mitochondrial DNA as particular cancer cargo, alongside with other cellular molecules such as RNA, proteins, and lipid rafts. All the previously mentioned biomolecules, that are pleiotropically functional in the receiver cell, contribute to the alteration of the tumor microenvironment, angiogenesis, metastasis, and immunity avoidance [101].

Exosomes are responsible for their decisive role in cancer spreading because of their mediation in DNA horizontal transfer, comprising specific differentiations attributed to tumors. These differentiations occur by transmitting DNA from cancer cells to healthy ones, henceforth reflecting the transformation of the receiver cell to a condition premalignant or malignant. Exosome-derived miRNAs may also be considered as candidate biomarkers for glioma, although additional scientific attempts are required to promote the investigation of clinically helpful biomarkers for gliomas [102].

In brain cancer, a resistant cancer type in immune cell recruitment, exosomes originated from DCs have been considered as a favorable treatment strategy against glioma, applied in a mice model, and this immune-related and exosome-based approach constituted an unconventional therapy for GBM [26]. Another noteworthy property of exosomes is that they can traverse through an uninjured BBB and anatomical compartments through transcytosis, which is the common strategy of multicellular organisms utilized to selectively transport vesicular macromolecules between two surroundings without altering their unique composition [103].

In contrast to the natural ones, exosomes derived from desired design and modifications could eliminate the restrictions derived from natural exosomes, such as lack of target recognition capability, insufficient half-life in circulation, and a small quantity of functional molecules. Similarly, exosomes profit concomitantly from their content in surface-decorated and encapsulated molecules [104].

Many attempts have been made to clarify the properties of TdEXs as sources of tumor antigens and immune adjuvants. Nevertheless, several statements have proposed TdEXs to be involved with pro-tumorigenic roles. The complicated functionalities of TdEXs must be investigated exhaustively, and an in-depth exploration into TdEX biogeneration and identification will expand their usefulness by permitting the TdEXs optimization reflecting on the alleviation of their adverse features and amplifying their potency [14].

## 3. Exosomes in the Diagnosis of Brain Cancer

Malignant brain tumors grow rapidly and invade surrounding healthy tissues. They can be distinguished in primary and metastatic or secondary brain tumors that originated in another part of the body and metastasize to the brain. Gliomas are primary brain cancer and are classified into four tumor grades (I–IV) according to World Health Organization (WHO) guidelines [105]. In grade IV, there is GBM, which is a very common and quite aggressive type in adults. It is reported that less than 5% survive in a 5-year period from the first diagnosis, despite the recent advances in treatment consisting of surgery, radiation and chemotherapy [106].

GBMs are significantly heterogeneous and have similar radiological features with other primary tumors, abscesses, and metastases, raising difficulties in their pathological diagnosis. So far, the diagnosis of GBM is performed by computed tomography (CT) or magnetic resonance imaging (MRI). However, clinically useful liquid diagnostic biomarkers are needed for early diagnosis, differentiation from other primary tumors and monitoring of the disease. Diagnostic biomarkers would be also extremely useful when surgery is not applicable, biopsy results are insufficient, and early GBM detection is needed in tumor recurrence.

Proteins and nucleic acids, especially RNA, are used as diagnostic and prognostic biomarkers for various neurodegenerative diseases, brain cancer and other disorders. A satisfactory biomarker should permit premature disease detection and, eventually, distinguish the evolution of normal conditions, prognosticating the reactions/response to treatment. Currently, several types of research have focalized on the whole source of biomarkers circulating in biofluids; however, it is notable that exosomes originated from biopsy fluids may possibly constitute a novel aspect of personalized medicine application.

Exosomes secreted from different kind of cells possess particular features, different composition, and actions on their selected target cells [79,80]. Furthermore, exosomes as might be expected exhibit the same features with their parental cells [81,82]. Specifically, their composition of genome and proteome should mirror the situation of parental cancerous cells that permit the recognition of quick alterations in the tumor cell condition and evolution [83]. The exploitation of these dominant exosomal features may be useful in several biomedical applications. Exosomes constitute an abundant source for feeding the discovery of biomarkers contained in the biofluids, which is suitable for the diagnosis of various diseases, including cancers and others [107,108]. Additionally, exosomes possess proteins that have biomarker potential for many cancers [109], and it is indisputably admittable that the premature discovery of a tumor is pivotal for an effective therapy [110].

Exosomes originating from cancer cells trigger tumor-advancement outcomes, liberating mainly proteins, including oncoproteins, growth factors, and various RNAs, such as messenger RNAs (mRNAs), miRNAs, and small-interfering RNA (siRNA), which intercede in particular signaling mechanisms and pathways associated with dysregulated cell growth [111]. It is noteworthy that exosomes secreted by cancer cells contain abundant tumor-specific molecules, approximately 100 times more than those existing in the original tumor cells [14,112]. Therefore, it is obvious that exosomes could amplify, through a non-invasive strategy, the synergistic diagnostic efficiency of biomolecules combination compared to a conventional diagnostic marker [113].

### 3.1. Exosomal miRNA Biomarkers in Brain Cancer

Recent studies show that brain tumor cells release a significant number of exosomes that cross the BBB and can be identified in the bloodstream of patients with malignant gliomas [114,115]. miRNAs, present in exosomes, are important biomarkers for the prognosis and identification of cancer due to the fact that they have both oncogene and tumor suppressor gene functions [86,116]. miRNAs are non-coding RNAs, around 20–22 nucleotides, that participate in the protein synthesis, cell growth, development, invasion, differentiation, and progression of cancers [117,118].

The exosomal miRNAs could be considered as promising biomarkers in GBM, the most aggressive primary intracranial tumor of the adult brain, since there are clinically used methodologies for the detection of miRNAs and well-established isolation protocols of exosomes from the peripheral blood. miRNAs provide information about the disease, its progression as well as the response to various treatments [116]. miRNAs can operate with a bilateral role either as contingent oncogenes or tumor suppressors in gliomas [119].

A study displayed that miR-181a, miR-181b, and miR-181c function as tumor suppressors in GBM and contribute to the complexity of the pathological progression of glioma [120,121]. Furthermore, several RNAs have been recognized as glioma-specific biomarkers including EGFR (EGFRvIII, epidermal growth factor receptor variant III), isocitrate dehydrogenase 1 (IDH1), a promising therapeutic target in selected cancers with a mutated version of the mitochondrial enzyme IDH1, and miR-21 [86,122].

MiR-21 is a familiar regulator of EGFR expression and has been found in the CSF of 100% of GBM patients, pointing out that may be considered a greatly sensitive marker for GBM detection. Moreover, the significance of miR-21 expression has also been associated with tumor-induced manipulation of the micro-surrounding during glioma cell invasion [123,124]. miR-21 affects cell proliferation through the phosphoinositide 3-kinase (PI3K), the phosphatase and tensin homolog (PTEN), Sprouty and the tumor suppressor gene tropomyosin 4 (TPM4). The reduction in apoptosis is achieved through the pro-apoptotic FAS ligand (FasL), the B-cell translocation gene 2 (BTG2), a member of the F-box subfamily 1 named F-box protein 11 (FBXO11) and the tissue inhibitor of metalloproteinases 3 (TIMP3). miRNA also affects angiogenesis and invasion through PTEN, TIMP3, and tropomyosin 1 (TPM1) [125]. A meta-analysis of recent studies covering various cancer types indicated that miR-21 isolated from exosomes could be used as a biomarker in cancer diagnosis, although more than one miRNA marker combined with other cancer antigens would detect cancer with more sensitivity and accuracy [125].

Santangelo and co-workers studied the expression levels of three exosome-associated miRNAs (miR-21, miR-124-3p, miR-222) in the serum of individuals with high- and low-grade gliomas (HGGs and LGGs) both before and after surgical resection. Their results, in combination with other studies [126], revealed that patients with glioma overexpressed miR-21, and only miR-21 could be successfully used as a non-invasive clinical biomarker [102]. The overexpression of miR-124-3p was also found, and its level was higher than miR-409-3p, miR-127-3p, and miR-181d-5p in glioma tissues, while it is rarely overexpressed in other cancers [127].

As Santangelo and co-workers showed, the combined miR-21, miR-222 and miR-124-3p expression was very effective in differentiating patients with HGG from patients with LGGs and healthy individuals, since their levels were significantly increased in HGGs. After surgical removal of the tumor, those levels were found to be significantly lower [102]. In a recent study of Catelan et al., they studied miR-21, miR-124-3p and miR-222 expression levels in exosomes isolated from patient’s serum with HGGs and brain metastases (BM) to validate miRNAs efficacy in the differential diagnosis. They also determined variations of miRNAs levels after surgery and/or BM treatment. The location of primary cancers of all patients with BMs was varied. MiR-21 expression was increased significantly in both HGGs and BMs compared with healthy controls, while miR-124-3p expression levels were found decreased in BMs and increased in HGGs. Cancer surgical removal resulted in a significant reduction in miR-21. Therefore, elevated levels of miR-21 and reduced levels of miR-124-3p in serum exosomes is characteristic molecular evidence of BMs [128].

Manterola and co-workers used gene chip technology to study the expression of miR-320, miR-574-3p and RNU6-1 (RNA, U6 small nuclear 1) in patients with malignant GBM [129]. They found that RNU6-1 alone or the combination of all three biomarkers was considerably correlated with the diagnosis of GBM. RNU6-1 is part of the splicing machinery, and it is synthetized by RNA polymerase III, which is negatively regulated by PTEN and retinoblastoma protein (Rb) tumor suppressor proteins [130,131]. Enhanced activity of RNU6-1 has been shown necessary for tumorigenesis [132]. Both miR-320 and miR-574-3p were found in serum or plasma and have important roles in different cancer types [133,134].

Exosomal sncRNAs, isolated from twelve patients with GBM, were unbiased deep sequenced and identified. In addition, various miRNAs (miR-23a, miR-30a, miR-221, and miR-451.6) were implicated in oncogenesis [135].

Seven of the twenty-six differentially expressed miRNAs were selected, and the expression levels of miR-182-5p, miR-328-3p, miR-339-5p, miR-340-5p, miR-485-3p, miR-486-5p, and miR-543 diagnose GBM with 91.7% accuracy. The diagnosis of GBM patients from healthy controls with 100.0% accuracy could be also achieved using only a combination of four miRNAs (miR-182-5p, miR-328-3p miR-485-3p, and miR-486-5p).

miR-182 was suggested as a marker of glioma tumorigenesis and progression with high expression in GBM tissues [136] and is associated with poor overall survival [137]. In addition, miR-486 overexpression promotes glioma aggressiveness in vitro and in vivo [138]. Exosomal miR-328 and miR-340 expression levels were reduced in GBM patients sera, while low levels of miRNA-485-5p and miR-543 were detected in tumor tissue [139,140,141] and are associated with poor outcome rates [139,140]. Decreased levels of miR-339 in GBM patients was shown to contribute to the immune evasion of GBM cells by modulating T cell responses [142].

Lan and co-workers correlated exosomal miR-210 levels and hypoxia in patients with glioma [143]. In hypoxia, tissues or organs receive inadequate oxygen, and, as a result, the hypoxia-inducible factors (HIFs) are abnormally activated and significantly regulate tumor signaling [144]. Therefore, hypoxia has been conceded as the main cause of poor prognosis in GBM patients [145].

Serum exosomal levels of miR-210 were increased in glioma patients compared with healthy ones. In addition, the miR-210 increase was proportional to glioma’s pathological grades. In LGGs patients (I–II), the expression of miR-210 was relatively lower than in HGG patients (III-IV). Furthermore, the expression levels of exosomal miR-210 were declined in GBM patients after surgery, and in cases where the primary tumor reoccurs, miR-210 levels re-increased [143].

In various cancer types, the overexpression of miR-210 is regulated in a hypoxia-inducible factor-1a (HIF-1α)-dependent manner [146,147]. This finding encouraged Lan and co-workers to examine serum exosomal miR-210 levels compared with Carbonic Anhydrase 9 (CA9) and HIF-1α levels in GBM patients. They revealed that all markers had high expression levels, concluding that miR-210 reflects hypoxia in patients with glioma and could be used as a hypoxia potential biomarker [143].

The exosomal miR-2276-5p was determined in glioma patients, and it was found that its expression was quite reduced compared with non-glioma patients. The lower expression of miR-2276-5p was also linked with a lower rate of survival [148]. In the same study, Sun and co-workers used bioinformatic tools for the prediction of miR-2276-5p possible targets. Among them, Ras-related protein 13 (RAB13) is overexpressed in patients with glioma, especially in HGG, and it was also correlated with poor survival.

The role of miR-2276-5p in glioma is still unknown, although it is known that all Rab and Rab-like proteins participate in the regulation of membrane transport [149], in exosome formation [150], and exosome secretion is adversely affected by their absence [151].

Exosomal miR-454-3p turned out to be a promising biomarker for the identification, grading and prognosis of gliomas [152]. It can be also used to estimate glioma progression and patient response to therapeutic strategies. Shao and co-workers revealed that one of miR-454-3p targets is Autophagy Related 12 (ATG12) and, as a consequence, cell proliferation is suppressed. Furthermore, invasion and autophagy are also reduced. The role of miR-454-3p in different types of cancer is diverse, and it is expected that the effect of glioma exosomal miR-454-3p in different tissues is variable.

### 3.2. Exosomal circRNA Biomarkers in Brain Cancer

CircRNAs have a covalently closed structure that confers them high tolerance to exonucleases. They are abundant, conserved, and stable molecules implicated in gene regulation [153,154]. CircRNAs participate in the regulation of the parental gene alternative splicing and function as peptide-coding RNAs or miRNA sponges [155,156]. CircRNAs are differentially expressed in various tissues and can be found in exosomes, plasma and saliva. Their function renders exosome circRNAs as a new and promising cancer biomarker applicable in various diseases [157,158]. Different expression levels of circRNAs have been also found in gliomas, while other circRNAs encoded peptides that participate in GBM tumorigenesis [159,160,161]. Li and co-workers determined all circRNAs in primary high-grade astrocytoma (HGA) cells and in exosomes isolated from them. A panel of exosome circRNAs was verified (hsa_circ_0002976, hsa_circ_0075828, hsa_circ_0003828) for the early HGA screening, while four tissue circRNAs (hsa_circ_0005019, hsa_circ_0000880, hsa_circ_0051680, hsa_circ_0006365) could be used for the prognosis of HGA [162].

### 3.3. Exosomal Gene Biomarkers in Brain Cancer

mRNA, miRNA and proteins are the most prevalent molecular constituents of exosomes, although some exosomes contain double-stranded DNA [163]. Based on this report, Yang and co-workers investigated GBM orthotopic xenografts and revealed that *p65* (also named *RELA*), *DNM3* (Dynamin 3) and *CD117* (KIT proto-oncogene receptor tyrosine kinase) expression levels increased, whereas *TP53*, *APC* (Adenomatous polyposis coli) and *PTEN* decreased in tumors and blood exosomes [164]. In a previous study, the same research team found that tumor cells were more aggressive when the tumor reoccurred. Therefore, they compared normal brain tissues and recurrent tumors and found that various genes were dysregulated in the latter. Specifically, the three most up regulated genes were *DNM3*, *LLGL2* (LLGL Scribble Cell Polarity Complex Component 2) and *p65*, while the three most down regulated genes were *TP53*, *PTEN* and *ST14* (ST14 transmembrane serine protease matriptase). In exosomes from xenografts bearing recurrent tumors, the most up regulated genes were *DNM3*, *ZEB1* (Zinc finger E-box binding homeobox 1) and *p65*, while the most down regulated genes were *ST14*, *GATA2* (GATA-binding factor 2) and *TP53*. In conclusion, they found that up regulation of *DNM3* and *p65* and the down regulation of *TP53* are similar in blood exosomes and brain tissues, both for primary and recurrent GBM, and could serve as GBM diagnostic markers [164].

The *EGFR* and its variant *EGFRvIII* are highly expressed in GBM tumors. In the study of Manda and co-workers (2018), the authors tried to establish a non-invasive method for the identification of GBM using *EGFRvIII* detection in serum exosomes [165]. They detected the *EGFRvIII* transcript in 39.5% of tumor tissue samples and in 44.7% of their paired serum exosomes. Co-expression of the wild-type *EGFR* and *EGFRvIII* was identified in 28.1% of biopsy tumors, while the *EGFRvIII* expression either in tissue or exosomes was associated with a small survival rate. Both the sensitivity and specificity of the established assay was around 80%.

However, in 12 cases, the *EGFRvIII* was identified in the exosomes and not identified in paired tissues. This may be attributed to the significant inter- and intra-tumoral heterogeneity of the high-grade glial tumors; as a result, the tissue sample was not representative of the patient’s health condition. In addition, the tumor tissue samples had considerable amounts of necrosis, which might influence the identification potency of EGFRvIII. In seven cases, *EGFRvIII* was detected in tissue samples and not detected in the exosomes [165].

Thus, the detection of additional biomarkers apart from *EGFRvIII* is necessary to complement medical imaging, improve the specificity of the diagnosis and add more value in liquid biopsy.

### 3.4. Exosomal Protein Biomarkers in Brain Cancer

Recent evidence supports that exosomes originating from cancer cells convey a functional protein named programmed death-ligand 1 (PD-L1) and inhibit immune responses [166]. PD-L1, also called cluster of differentiation 274 (CD274) or B7 homolog 1 (B7-H1), is a type I transmembrane protein that is widely expressed in various cell types, including tumor cells, can mainly be expressed in immune advantageous regions, including the brain and others, and has been assumed to be involved in the suppression of the adaptive arm of immune systems during particular events [167]. This protein functions like a sort of “brake” to maintain the body’s immune responses below monitoring. PD-L1 exists in high content in some cancer cells, but it may be present in some normal cells in lower concentration. When PD-L1 is attached to the T cells-localized programmed death-1 (PD-1) protein, it prevents them from killing the PD-L1-containing cells, such as cancer cells. PD-L1, which blocks immune attacks on cancer cells, is recorded as a poor prognostic biomarker [168].

Favorable prognostic biomarkers carried within GBM-derived exosomes were considered the MGMT (O(6)-methylguanine DNA methyltransferase) and APNG (alkylpurine-DNA-N-glycosylase), which are the crucial enzymes able to repair TMZ-induced DNA damage, and their levels in tissues are inversely correlated with the therapy efficacy, while their expression is correlated with chemo-resistance [169].

Chun and co-workers performed a proteomic analysis of exosomes in the U-87MG, a GBM cell line, and identified proteome changes regarding to low temperature [170]. Among these proteins in the U-87MG exosome, myosin heavy chain 1, keratin, coatomer protein complex subunit, annexin A5, and collagen type VI and type I are abundant structural proteins in cells and the ECM. Collagen is the major component of the tumor microenvironment and promotes cell growth and migration. Collagen type I binds to its receptors, thus promoting tumor cell proliferation and epithelial–mesenchymal transition, while collagen type VI forms tissue microfibrils [171]. Coatomer is a precursor of coat protein I (COP I) that regulates Golgi apparatus vesicle formation, Golgi budding and vesicular trafficking [172]. Annexin A5 is a typical member of the annexin protein family and has high specificity for calcium and phosphatidylserine. The binding of phosphatidylserine is a main signal for phagocytes of apoptotic and necrotic cells. Annexin A5 was found to interact with polycystin-1, a metastasis-related protein that induces the suppression of tumor metastasis [173]. Therefore, the identification of these proteins in exosomes could be used for the detection of GBM.

Annexin A1 was also identified as a potential GBM biomarker in the study of Naryzhny and co-workers (2020) [174]. They presented a proteomic analysis of exosomes obtained from five GBM cell lines (A172–536, Glia-Tr–367, Glia-Sh–373, Glia-R–385 and Glia-L–482), and the 133 most frequent exosomal proteins were determined [174]. Eleven proteins from this list are present in all exosome samples and were studied. These proteins are annexin A1 (ANXA1), annexin A2 (ANXA2), pyruvate kinase M1/M2 (PKM), triosephosphate isomerase (TPIS), alpha-enolase (ENOA), vimentin (VIME), transitional endoplasmic reticulum ATPase (TERA), peroxiredoxin-1 (PRDX1), glyceraldehyde-3-phosphate dehydrogenase (G3P), heat shock protein 90-beta (HS90B), and 14-3-3 protein epsilon (14-3-3E). Two more proteins, cofilin (COF1) and nucleophosmin (NPM), are present in four exosome samples [174].

Additionally, the DNA-dependent protein kinase catalytic subunit (PRKDC) and the major vault protein (MVP) were found to contribute to the development of GBs and treatment resistance. PRKDC is involved in the repair mechanism of double-strand breaks causing radio-resistance [175]. Vasolin-containing protein (VCP) regulates PRKDC degradation and was also found in the exosome samples [176]. MVP is associated with nuclear pores, participates in nuclear–cytoplasmic transport regulation and reveals resistance to certain drugs [177,178]. The MVP gene is a target of the transcription factor GLI family zinc finger 1 (GLI1) [179], which is abnormally active in glioma lines [180]. The above-mentioned proteins can be also characterized as “exosomal GBM biomarkers”.

In the study of Cumba Garcia and co-workers, cytokines were determined in plasma-derived exosomes from patients with GBM and healthy individuals. They demonstrated that Interleukin-3 (IL-3), Interleukin-10 (IL-10) and Interferon-gamma (IFN-g) were significantly decreased in GBM [181]. They also found decreased levels of the co-stimulatory proteins B7-1, B7-2, and Inducible co-stimulator ligand (ICOSL) in GBM. However, highly expressed levels of the PD-L1 in GBM and healthy individuals indicated that PDL is not an appropriate biomarker of malignancy, as also mentioned by Litak et al. [168].

A novel and very promising diagnostic tool for GBM was developed by Qiu and co-workers [182]. They took advantage of the fact that the EGFRvIII mutant protein is related with glioma diagnosis and prediction, and it is located on the U251 glioma cells membrane as well as their exosomes [183]. Because of the high affinity between biotin and the TiN surface, they immobilized biotinylated anti-EGFRvIII antibody on the TiN surface. The anti-EGFRvIII functionalized titanium nitride (TiN) to produce Biotinylated Antibody-Functionalized Titanium Nitride (BAF-TiN) biosensors revealed good sensitivity and selectivity toward GMs-derived exosomes. The detection limit of exosome concentrations ranges from 0.0005 to 1000 μg/mL, while the size of exosomes ranges between 30 and 150 nm. The detection limit for the exosomal membrane proteins studied was 2.75 × 10^−3^ μg/mL for EGFRvIII and 4.29 × 10^−3^ μg/mL for CD63, an exosome marker [182]. The BAF-TiN biosensors showed fast and effective functionalization and exhibited excellent sensitivity. They were tested in the detection of serum exosomes isolated from mouse and found that BAF-TiN biosensors were capable of determining exosomes and exosomal proteins in real biological fluids.

**Figure 3:**
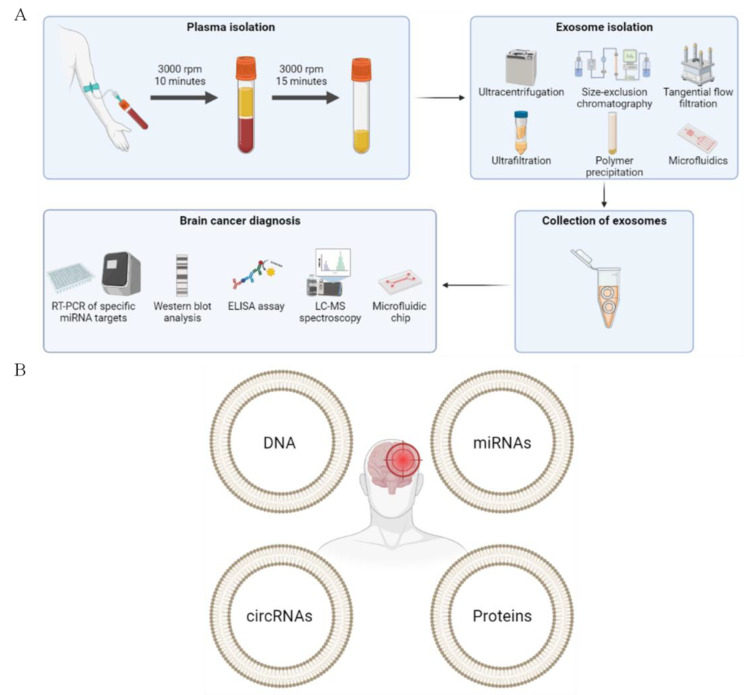
(**A**) Steps to brain cancer diagnosis utilizing exosomes. (**B**) Exosomal biomarker groups for brain cancer diagnosis. Created with Biorender.com.

Therefore, the BAF-TiN biosensor is beneficial for the quantification of protein biomarkers derived from glioma exosomes in a fast and simultaneous way. They could be applied in many biomedical fields, such as diagnosis (Figure 3), pathogen detection, immunotherapy and the pharmaceutical industry.

## 4. Exosomes in the Treatment of Brain Cancer

The field of brain cancer treatment faces many challenges as a consequence of the lack of selectivity, effectiveness, BBB penetration, postoperative recurrence, and drug resistance caused by conventional therapeutic protocols [7,184]. The continuously increasing knowledge of molecular techniques in parallel to advances in brain tumor biology has notably changed cancer management during the past 10 years. However, paramount obstacles attributed to tumor heterogeneity still hinder the design of precise and efficient therapies [185].

Nanotechnology has the potential to overcome such barriers and additionally confers a comparative advantage to conventional therapeutic protocols since it offers personalized treatment options [186]. Exosomes represent an ideal nano-scale carrier of antineoplastic agents due to their compatible nature with human organisms, increased stability, permeability, insignificant possibility of eliciting an immune response, and extended bloodstream presence, as well as elevated encapsulating amplitude and efficiency [187,188]. Although the field around the study of exosomes in cancer has drastically changed, the effectiveness of exosomes in cancer detection or treatment has not been particularly noteworthy. Yet, research on exosomes has revealed that they could be utilized as a revolutionary weapon in cancer diagnosis and therapy with a continuous increase in the extensive research conducted in this scientific discipline [2].

Εxosomes exhibit excellent properties as drug carriers. Possessing supremacy over other formulations, they are actively utilized in various therapeutic strategies against tumors either to induce the expression of various active biomolecules or to deliver biomolecules and drugs. Moreover, they have recently been mentioned in clinical trials [189]. A recent review provides fundamental insights and focuses on epitomizing the exosomal qualitative features to be employed as drug vehicle carriers in various techniques for effective drug loading, strategies for target recognition, and in vivo and in vitro tracing methods [19]. Owing to the appropriate size and properties of exosomes in combination with their therapeutic benefits and unidentified or incomprehensible still functions, they prompted the interest of scientists in evolving drug delivery systems based on exosomes. The review is focused on the exosomal properties scrutinization, including structure, and characteristics, as well as their carrier functions for diagnosing and treating disease [19].

Collecting all the considerable research data on exosomal exploitation as vehicles for drug delivery, it is concluded that they are capable of being loaded with a range of curative agents for the treatment of various diseases. It is notable that based on the exosomes’ properties exclusively, it could not possibly be managed to achieve a successful transfer of cargos and, ideally, drug accumulation toward the damaged/defective tissues. Whether the exosomal drug loading system is located far away from the target tissue, the treatment factor concentration will be diminished as it becomes metabolized and eliminated by the body. This can lead to a reduced or insufficient amount of the drug reaching the target tissue, which may result in a failure to achieve the desired therapeutic outcome. Apart from that, it is possible to notice non-selective drug accumulation, henceforth triggering undesired toxic side effects. Therefore, a considerable volume of scientific reports point out that exosomes have been found to employ various tactics to achieve directed targeting and improve the therapeutic outcome.

Although exosomes have great potential to be utilized as brain drug delivery vehicles, their essential roles are related to brain well-being, for instance, brain homeostasis and improvement of exosomal curative ability, which is expected to be enhanced in the future with various engineered methodologies that will aim to amplify the possibilities of target recognition, organ or site bio-distribution, and an efficacious BBB passage across. In addition, the provocation of achieving the necessary quantity of exosomes to proceed for clinical applications could also be increased by employing relevant biotechnology approaches [24]. Keeping in mind the desired properties of an efficacious drug delivery, exosomes are likely candidates for clinical translation [24].

Furthermore, exosome membrane hybridization has recently attracted interest for utilization as a drug delivery system and also opened a field for the specific targeting of tissues in comparison to naïve exosomes [190]. This advanced and original approach covers nanocarrier systems with appropriate cell membranes able to target the desired tissues, especially neoplastic tissues due to their tropism cell properties, whose phenomenon of oriented movement is directed toward the source of the stimulus that acts with greater intensity from one direction to the other [191].

Overall, while exosomes hold promise as a tool for cancer treatment, there are several challenges that need to be addressed to enable their effective clinical development, and these issues were reviewed recently [14]. Their transport ability of therapeutic constituents including siRNAs, miRNAs, membrane-related proteins, and chemotherapeutic agents render exosomes as the principal candidate delivery tool of biomolecules for cancer treatment. Moreover, due to their potential to create an appropriate micro-surrounding for the efficient functionality of immunoregulatory factors, exosomes-hosting selected bioactive components have been bioengineered accordingly as tumor immune-therapies able to effectually stimulate the cancer immunity cycle in each stage to successfully provoke cancer-specific immunity [14].

**Figure 4:**
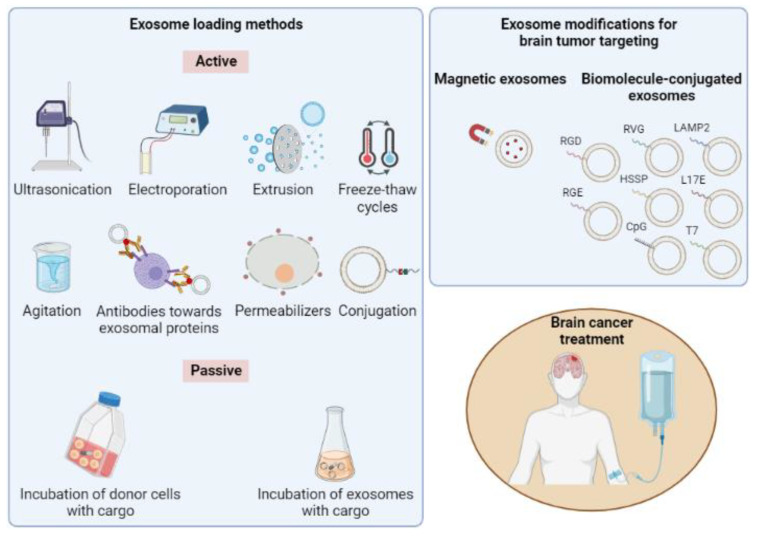
A representative scheme of exosome loading and modification methods. Created with Biorender.com.

The natural barrier provided by the lipid bilayer membrane of exosome vesicles protects their cargo from degradation in the bloodstream; however, the same membrane and the intrinsic contents of exosomes can present difficulties in loading drugs into them [192]. Two main approaches, active and passive, are typically utilized to incorporate cargo into exosomes [193]. In more detail, the active loading approach includes sonication [194,195], extrusion [194,195], freeze and thaw cycles [194,195], electroporation [194,195], membrane permeabilizers [194,195], chemical methods for direct conjugation [194], antibodies against exosomal proteins [194] and agitation [196]. On the other hand, the passive cargo loading method involves the incubation of drugs either with exosomes or with donor cells [194,195]. An illustrative scheme of exosome loading and modification methods is given below (Figure 4).

Although there is a plethora of exosome classification methods and approaches, exosomes are mainly classified based on their source; however, it is also feasible to be divided into natural and engineered, according to the work of Liu and his colleagues [197]. By referring to the term source, we consider the cell line from which exosomes were produced or isolated.

### 4.1. Mesenchymal Stem Cells-Derived Exosomes

MSCs have the ability to relocate to glioma cells and exert their antitumor effects by releasing cytotoxic agents. The same cells hold excessive regenerative capacity as well as reinforce the functions of immune system. A therapeutic approach utilizing MSCs involves cell replacement and the release of different vesicles, such as exosomes. Specifically, exosomes obtained from MSCs are a highly effective treatment option for several illnesses, including brain cancer, and they hold great promise for regenerative medicine, both in terms of cell-based and cell-free therapies [198]. Engineered MSC-derived exosomes diminished resistance to conventional therapies, such as radio-, chemo-, and anti-angiogenesis therapy [198].

An important step in the field of GBM treatment was accomplished by Tibensky and his team, who investigated the effectiveness of yeast cytosine deaminase::uracilphosphoribosyl transferase (yCD::UPRT) MSCs-engineered exosomes along with 5-fluorocytosine (5-FC). Their results reflected the potential of these exosomes to retard the growth of C6 GBM cells both in vitro and in vivo, when administered intranasally, intraperitoneally, or subcutaneously [199]. The superiority of their approach is the direct conversion of the moderately harmless agent 5-FC into the extremely potent anticancer drug 5-fluorouracil (5-FU) inside the tumor mass, therefore hampering systemic toxicities and enhancing the targeting ability [199]. In order to investigate the delivery of synthetic miRNA to glioma cells and glioma stem cells, Lee and his colleagues utilized mesenchymal stem cells obtained from various sources, including bone marrow, adipose tissue, placenta, and umbilical cord. Although the aforementioned cell lines express these miRNAs at low levels, the administration of miR-124 and miR-145, in particular, was explored. The delivery of these miRNAs led to a notable decrease in the reported target genes’ luciferase activity. Moreover, glioma stem cells’ ability to be subjected to self-renewal as well as glioma cells’ reduced migration rate were both noted. Additionally, in vivo results were conducted to U87-derived xenografts, and the team found that the delivered Cy3-miR-124 penetrated the above-mentioned xenografts and downregulated the expression of cyclin-dependent kinase 6 (CDK6), which is a miR-124 target gene. The findings from this research also shed light on brand-new strategies for the targeted shuttle of antineoplastic miRNAs in GBM that may be used for other types of tumors and possibly replace current therapies in the future [200].

Furthermore, the administration of effective anti-miR-9 through exosomes obtained from MSCs to GBM resulted in increased responsiveness to chemotherapy, as indicated by augmented cell cytotoxicity and elevated apoptosis [201]. The most likely reason for that phenomenon is the involvement of miR-9 in the inducement of P-glycoprotein expression, which represents an important drug efflux transporter on the cell surface. Yan and his colleagues transfected bone mesenchymal stem cells (BMSC) with miR-512-5p, and the exosomes collected inhibited GBM cell proliferation and led to cell cycle arrest by targeting and suppressing Jagged Canonical Notch Ligand 1 (JAG1) [202]. MiR-199a-overexpressing MSCs isolated exosomes have successfully been utilized to abort glioma progression through the downregulation of ArfGAP with GTPase Domain, Ankyrin Repeat and PH Domain 2 (AGAP2). As a result, a suppression in the cellular growth, infiltration, and migrative behavior of glioma cells was observed, whereas the chemosensitivity to TMZ was restored and the tumor growth was repressed in vivo [203]. The transfer of MiR-29a-3p using exosomes, obtained from human MSCs that were engineered, had a beneficial impact on the suppression of tumors by decreasing the movement and formation of vasculogenic mimicry in glioma cells both in vitro and in vivo [204]. Next, exosomal miR-133b obtained from MSCs attenuated glioma development via aborting the Wnt/β-catenin signaling pathway through Enhancer of Zeste 2 Polycomb Repressive Complex 2 Subunit (EZH2) expression inhibition and, as a result, suppressed cellular growth, infiltration, and migrative behavior as well as it could be utilized of therapeutic interest [205]. In addition, there is plenty of evidence that MiR-584 serves as a tumor suppressor in many neoplasms by attaching to the 3’ untranslated region (UTR) of CYP2J2. As a result, the introduction of miRNA-584-5p into U87 cells led to the substantial suppression of Cytochrome P450 2J2 isoform (CYP2J2) expression, which subsequently caused a decline in the rate of proliferation and invasiveness of glioma cells. Indeed, U87-treated cells with exosomes originating from MSCs that were transfected with miRNA-584 displayed also reduced cell propagation and migrative behavior in vitro, while in vivo data in athymic mice demonstrated an attenuation of malignant gliomas development [206].

Marrow stromal cell (MaSC) exosomes have the potential to be exploited as a transport system of antineoplastic miRNAs, such as miR-146b, and the consequent intra-tumor injection of the aforementioned engineered vehicles pointedly abolished glioma xenograft development in a rat model of a primary brain tumor [207]. The effect of MSC-isolated exosomes on C6 cells in vitro was delineated by Parsaei and his research team, where they isolated and described exosomes from MSCs derived from the bone marrow of rats (rBMMSCs) following the incubation of various concentrations of the above-mentioned exosomes with C6 cells. Their results confirmed apoptosis induction in C6 cells, while a direct relationship was noted between the concentration of exosomes and their toxicity [208]. Additionally, Rehman and his team investigated allogeneic bone marrow mesenchymal stem cells-derived exosomes (BMSCExo) with heme oxygenase-1 (HMOX1) specific short peptide (HSSP) to serve as a TMZ and small interfering RNA (siRNA) nanovehicle in the treatment of GBM that is resistant to TMZ. Their experimental protocol revealed both in a laboratory setting and in live organisms that BMSCExo exhibited a remarkable ability to target TMZ-resistant GBM owing to the overexpression of HMOX1 in the resistant cells. Additionally, the delivery of siRNA targeted to signal transducer and activator of transcription 3 (STAT3) in the TMZ-resistant glioma cells restored TMZ sensitivity. These findings suggest that BMSCExo decorated with HSSP-HMOX1 could be a promising therapeutic option for TMZ-resistant GBM and have the potential to lead to apoptotic death of U251-TR cells through the STAT3–MGMT signaling pathway [209]. In addition, a potent anti-glioma agent against Glioblastoma Stem Cells (GSCs) was revealed to be miR-124a. Specifically, miR-124a engineered exosomes isolated from BMMSCs represented an ideal weapon in GBM treatment as exemplified by both in vitro assays, in which an important reduction in the viability and colony-forming ability of GSCs was recorded, and in vivo gold-standard animal models through Forkhead box protein A2 (FOXA2) negative regulation that strongly affects tumor growth and survival [210].

Not only do cargo-loaded exosomes display anticancer activities, but they also may confer anti-angiogenic capability. Specifically, a new perspective in the treatment of GBM was revealed through the work of Valipour et al. [211]. Following the seclusion and identification of exosomes produced from mesenchymal stem cells of endometrial origin (hEnMSCs exosomes), the team exploited them as a novel nanoplatform for loading atorvastatin to investigate its potential in inducing apoptogenic and angiostatic effects on U87 GBM spheroids 3D that were co-cultured with human endothelial cells of umbilical vein origin (HUVECs). Their findings indicated that the concentrations of 5 and 10 μM of non-encapsulated atorvastatin and atorvastatin-encapsulated hEnMSCs exosomes (AtoEXOs) displayed substantial proliferation repression compared to other concentrations. Additionally, a reduction in HUVEC migration and a noteworthy reduction in vascular endothelial growth factor (VEGF) release, as well as an increase in the Bax/Bcl-2 expression ratio in U87 spheroids 3D co-cultured with HUVECs, were observed, particularly for the 10 μM AtoEXOs in comparison with the remaining cell groups. These results suggest that AtoEXOs foster apoptosis and prevent angiogenesis in U87 spheroids 3D co-cultured with HUVEC cells [211]. The same drug, atorvastatin, was also enfolded in exosomes isolated from human endometrial stem cells (hEnSCs) and, consequently, was evaluated regarding the tumor growth-suppressive effects of a 3D GBM model. Their results demonstrated a strong inhibition of tumor growth and inducement of glial cell death [212].

The discovery of ferroptosis opened new therapeutic directions for GBM therapy. An innovative curative platform for GBM therapy that utilized human mesenchymal stromal cells (hMSCs), engineered exosomes with magnetic NPs possessing enhanced BBB permeation ability and enveloping characteristics of glutathione peroxidase 4 (GPX4) siRNA as well as Brequinar (BQR), a dihydroorotate dehydrogenase (DHODH) inhibitor, was developed. This platform can be magnetically localized in the brain, and angiopep-2 peptide-modified engineered exosomes induced endocytosis, granting the NPs the ability to efficiently permeate the BBB and reach GBM cells through recognition of the LRP-1 receptor. The unique combination of magnetic targeting features and drug delivery properties of magnetic NPs, along with the GPX4 siRNA and BQR, rendered this nanosystem a promising candidate for GBM therapy, as it was displayed by in vitro and in vivo experiments. As a result, it could be a bright solution for the treatment of GBM [213]. Moreover, the role of hMSC-isolated exosomes incubated with miR-375 was studied, and the results displayed significant inhibition in cell propagation, migration, and infiltration ability as well as apoptosis induction through targeting solute carrier family 31, member 1 (SLC31A1), since the lower expression of miR-375 in glioma cells was restored [214]. Their findings were also confirmed by in vivo experimental protocols due to the repression of xenograft tumor in nude mice and, consequently, it could be exploited therapeutically [214].

The potential of human umbilical cord (hUC-MSCs)-derived exosomes was delineated by Hao and his colleagues [215]. Firstly, they co-cultured hUC-MSCs with U87 cells in order to examine the effects of hUC-MSC exosomes release on U87 cell propagation, and their data confirmed an inhibition of cell proliferation and apoptosis inducement [215]. Secondly, they identified that long non-coding RNA (lncRNA) phosphatase and tensin homolog pseudogene 1 (PTENP1) could be embedded into exosomes of hUC-MSCs and delivered to U87 cells. Thus, PTENP1 protected PTEN from degradation through sponging miR-10a-5p, and, consequently, suppressed the malignant phenotype of glioma U87 cells [215].

Another example in the management of brain cancer is exosomes isolated from MSCs, which are conjugated with superparamagnetic iron oxide NPs (Ex-SPIONs) and loaded with doxorubicin (DOX). Considering SPIONs’ magnetic properties, the targeted transfer of DOX-loaded Ex-SPIONs was established in rat brains and displayed the appropriate drug loading efficiency as well as enhanced cytotoxic effect against cancer cells. This system comprised potent and docile magnetic nanoplatforms of great bioaffinity to transfer drugs across intact biological obstacles, for instance BBB [216].

The efficacy of arginylglycylaspartic acid (RGD)-polypeptide-engineered EVs in delivering loaded cargo to GBM cells is a widely established phenomenon. Various studies have shown that incorporating an RGD peptide to exosomes’ surface as a ligand to GBM cell surface proteins and integrins enhances the internalization of EVs by GBM cells, resulting in improved drug delivery effectiveness, regardless of the type of cargo [217,218,219]. A recent literature review published by Ludwig et al. [220] summarized the crucial role played by different types of integrins in cancer, contributing to a wide range of cellular processes, such as tumor development, migration capacity, elevated motility and infiltration of neighboring tissues, and enhanced formation of blood vessels. Among them, αvβ3 and αvβ5 are overexpressed in GBM, whereas they are not found in non-cancerous brain tissue [218]. However, it is important to mention that this method is not the only one specific to GBM cells, as RGD peptides could be recognized from other cell types as well [218]. In addition, the effectiveness of drug administration and targeting to the brain was improved through engineered expression in the surface of EVs of brain-specific rabies viral glycoprotein (RVG) or internalizing RGD peptide (CRGDKGPDC), along with lysosomal-associated membrane glycoprotein-2b (Lamp-2b), as shown by different research teams [221,222].

Exosomes produced from engineered MSCs harboring an anti-EGFRvIII (ab139) antibody on their surface, while enveloping two pro-apoptotic gene therapy agents, such as cytosine deaminase (CDA) along with 5-FC administration and miR-34a, were extensively characterized and delivered to EGFRvIII+ GBM cells. The results indicated an enhanced apoptotic rate in U87EGFRviii cells compared with that for U87 cells, demonstrating selectivity, while the simultaneous delivery of the aforementioned factors succeeded in increased apoptosis through the intelligent mechanism described above, thus pointing out the effectiveness of targeting and combination therapy [223]. Bispecific antibodies targeting EGFRvIII and CD133 may be effective in treating GBM, according to research in mice and patient samples. In more detail, samples obtained from GBM patients usually include coexpressed CD133 and EGFRvIII proteins. In a mouse model of GBM, intracranially implanted cells that coexpressed CD133 and EGFRvIII, seem to increase tumor growth more than only CD133+ cells. A bispecific antibody that targets CD133 and EGFRvIII decreased tumor development and improved survival in the mouse model when compared to antibodies that only target either protein or IgG [224].

The fibroblast growth factor receptor 3 (FGFR3)-transforming acidic coiled-coil containing protein 3 (TACC3) fusion gene (F3-T3) has been characterized as a driver in gliomagenesis, and small-molecule inhibitors for targeting the produced protein have not been discovered until today. For this reason, different siRNAs targeting the aforementioned gene were designed, and MSCs-isolated exosomes were electroporated with the siRNAs. It was shown that F3-T3 depletion led to a diminished viability of cells and augmented survival in glioma-bearing mice without toxicity to adjacent normal tissues and without affecting the wild-type FGFR3 or TACC3 [225].

### 4.2. Embryonic Stem Cells-Derived Exosomes

Embryonic stem cells display great promise to be an attractive curative candidate in the treatment of aging-associated diseases such as cancer, relying on their distinct ability to proliferate endlessly, pluripotency, and an inherent barrier to aging [226]. Enhanced GBM cell targeting was achieved by the use of Embryonic Stem Cells (ESCs)-derived exosomes, loading with paclitaxel (PTX) as well as modified with Cyclo (Arginine-Glycine-Aspartic-D-Tyrosine-Lysine) peptide (c(RGDyK)) for having great binding capacity with αvβ3 integrin receptors. It was demonstrated that the above-mentioned exosomes improved the curative effects of PTX through optimized targeting [219].

### 4.3. Neural Stem Cells-Derived Exosomes

The pluripotent stem cells that are only located in the central nervous system are called neural stem cells (NSCs) and are characterized by great self-renewal capacity and the capability to differentiate into astrocytes and oligodendrocytes as well as neurons [227].

The exosomal transfer of specific miRNA cargos could enhance chemosensitivity to TMZ in drug-resistant GBM. Adamus and his colleagues exploited NSCs to transport antisense oligonucleotides (ASOs) targeting STAT3 conjugated with CpG that experience uptake via scavenger receptors, such as Toll-like receptor 9 (TLR-9), into the glioma microenvironment through exosomes compared to native NSC exosomes. Their results pointed out the stimulation of the immunogenicity of human dendritic cells or mouse macrophages and the induction of the nuclear factor kB (NF-kB) signaling pathway as well as Interleukin-12 (IL-12) generation. The same team confirmed that the accumulation of NSC-mediated transfer enhanced oligonucleotide delivery from a remote inoculation site into the glioma microenvironment in comparison with non-encapsulated oligonucleotides by using orthotopic GL261 tumors as well as inhibiting the growth of the aforementioned tumors [228]. The exploitation of NSCs-derived exosomes was also investigated by Qian and his colleagues. More specifically, they evaluated the transfer of miR-124-3p to glioma cells by NSC-derived exosomes in vitro and in vivo and observed a substantial repression of glioma cell propagation, migration, and infiltration as well as flotillin 2 (FLOT2) expression. In addition, they confirmed their therapeutic effects in a mouse tumor xenograft model of glioma with the same exceptional results, indicating the development of a possible strategy for the treatment of glioma [229].

### 4.4. Macrophage-Derived Exosomes

The innate immune system’s macrophages are a type of white blood cell that ingests pathogens such as cancer cells, bacteria, cellular debris, and foreign objects that lack proteins specific to healthy body cells [230].

A smart theranostic platform with efficient chemo/gene/photothermal therapy was introduced by Wang and his colleagues, where they isolated exosomes from biotin-labeled THP-1 induced cells into macrophages, loaded them with DOX, and functionalized them with polydopamine (PDA)-coated magnetic Fe_3_O_4_ NPs (Fe_3_O_4_@PDA). After magnetic navigation to the neoplastic tissue, the application of near-infrared radiation (NIR) prompted hyperthermia to the surrounding tissue and activated cargos liberation that aids miR-21 for both imaging and gene silencing with a significantly high apoptotic effect in cancer cells [231]. An analogous approach was followed by a research team, where they loaded NPs made from SPIONs and the diarylheptanoid agent, Cur into exosomes isolated from the Raw264.7 macrophage cell line, and afterward, modified exosomes’ membrane with neuropilin-1-targeted peptide (RGERPPR, RGE). The subsequent administration to GBM cell lines and orthotopic glioma models exhibited targeted imaging and treatment of that neoplasm, while side effects were diminished [232].

Another ‘’intelligent’’ nanoplatform based on sonodynamic therapy (SDT) was designed by Wu and his colleagues, where the enzyme catalase (CAT) was encapsulated into silica NPs for alleviating tumor oxygen deprivation conditions and, simultaneously, loaded with the sonosensitizer indocyanine green (ICG). To further increase BBB penetration, the nanostructure moiety is externally coated with AS1411 aptamer-modified macrophage exosomes. The produced NPs exploited the redox signaling capacity of cancer cells, generated O_2_ to alleviate tumor low-oxygen environment, and exhibited good biocompatibility as well as long circulation time underlining the potential of that nanoplatform for GBM therapy [233]. It is worth noting that the administration of that nanostructure significantly inhibited the tumor metastasis of GBM as well as displayed negligible toxicity to normal cells and tissues, thus underlining the biocompatibility of that type of engineered exosomes [233]. A precise boron neutron capture therapy (BNCT) with exosome-coated ^10^B carbon dots using D-glucose and boron phenylalanine (BPA) was introduced by Li and his colleagues with excellent results in a murine model of glioma in situ and 100% mice survival ratio, and the end of the experiment was recorded [234]. In parallel to spectacular results, they proved the doping of boron in the boron carbon dots (BCDs), followed by the encapsulation of BCDs with macrophage exosomes, and demonstrated the ability of the aforementioned engineered exosomes to pass through the BBB as well as to accumulate in tumor tissue, as shown by inductively coupled plasma mass spectrometry (ICP-MS) and fluorescence experiments [234]. Indeed, fluorescence imaging displayed stronger and for an extended period of time the gathering of BCD–Exos in comparison with free BCDs in the brain neoplasm of the murine model [234].

Similarly, Liang and his colleagues constructed dual-receptor-specific exosomes, isolated from the THP-1 cell line medium, as shuttles loaded with TMZ and O^6^-benzylguanine (BG) for eliminating GBM that is resistant to TMZ. Their exosomes managed to display strong cargo-protective capacity and effectively penetrate the BBB as well as accumulate in tumor sites and induce proliferation inhibition of U87MG and GSCs in vitro [235]. The utilization of macrophage exosomes functionalized with different types of cargo conferred a ray of hope in pediatric patients with diffuse intrinsic pontine glioma (DIPG). The research team that studied this rare cancer created a bioimitative nanodrug transfer system, which can efficiently co-administer Panobinostat, a non-selective histone deacetylase inhibitor and protein phosphatase 1D (PPM1D)-siRNA complexed into positively charged micelles with great drug loading efficiency. Afterward, the micelles were encapsulated by surface-modified exosomal coatings to facilitate the permeation of BBB and, consequently, targeted DIPG neoplasms. Their experimental protocols confirmed the efficient Panobinostat and PPM1D-siRNA delivery, while DIPG cell proliferation was significantly inhibited [236]. Moreover, a strong inhibition in cell propagation and invasion of glioma cells was displayed by circRNA BTG (circBTG2) anti-proliferation factor 2-loaded exosomes isolated from RBP-J overexpressing macrophages through miR-25-3p/PTEN axis [237]. Additionally, M2 macrophage-derived exosomes containing miR-15a and miR-92a inhibited the malignant behavior of glioma cells and, especially, migration and invasion through the PI3K/AKT/mTOR signaling axis by targeting Cyclin D1 (CCND1) and Ras-associated protein 1B (RAP1B), respectively [238].

Additionally, Bai’s team compared and examined the therapeutic potential of exosomes loaded with DOX, labeled with RVG-15 peptide for enhanced brain-targeting capacity and revealed that both macrophage-derived and blood-serum-derived exosomes could effectively deliver DOX into the tumor with the assistance of focused ultrasound (FUS). Their work resulted in the suppression of tumor growth either in vitro or in vivo [239].

### 4.5. Glioma Cells-Derived Exosomes

Liu and his colleagues synthesized, characterized, and assessed hydroxychloroquine (HCQ) encapsulated zinc sulfide (ZnS) NPs (HCQ@ZnS) for the therapy of GBM both in vitro and in vivo. The research team isolated exosomes from the cell cultivation media of human U87 GBM spheroids, characterized them and encapsulated HCQ@ZnS NPs in their core as well as attached an iRGD peptide in order to increase cellular uptake. Their study revealed remarkable permeation across the BBB and the precise targeting of cancer cells in an orthotopic murine GBM model. In more detail, ZnS acted as a photoactivator that generates reactive oxygen species (ROS) in GBM cells, which causes severe damage to organelles. On the other hand, HCQ was observed to inhibit autophagy, which can lead to the accumulation of damaged organelles resulting from excessive ROS production. As a result, the hybrid exosomes were able to guide the antineoplastic effects of HCQ and ZnS under light illumination, achieving substantial selective damage to GBM cells both in vitro and in vivo [217]. An analogous scientific perspective was adopted by Fan and his colleagues, where his team isolated exosomes from U87MG cells, loaded them with ICG, and attached in their surface the RGERPPR peptide (RGE). The new carrier showed significant abolishment of tumor growth in vivo through the inducement of apoptotic activity after laser irradiation [240].

TMZ resistance has been studied by various research teams, such as Wang’s team, where they designed reassembly exosomes (R-EXO) obtained from homologous glioma cells to encapsulate TMZ and dihydrotanshinone (DHT) for reversing resistance to TMZ and augmenting lesions-targeted drug transfer. It is worth mentioning that DHT can conquer the drug resistance originated by TMZ and displays excellent curative outcomes on glioma. Their results included desirable BBB-penetrating ability and potential anticancer activity with vanquishing TMZ resistance and activating immunologic reaction for both in vitro and in vivo experiments [241]. In more detail, miR-151a exosome encapsulation eliminated the chemoresistance propagation that was directed by TMZ-resistant exosomes and, consequently, promoted chemosensitivity to TMZ for in vitro as well as in vivo experiments [242]. Subsequently, exosomes derived from GBM cells and loaded with the anticancer medication selumetinib demonstrated potent antitumor effects on U87MG cells and were not harmful to normal brain cells. These findings, in conjunction with the lack of toxicity to the liver and kidneys in vivo, suggested that U87-selumetinib exosomes may be a viable therapeutic alternative for treating GBM [243].

Simultaneously, when PTX was loaded in exosomes isolated from cell culture media of U87 and researchers investigated U87 and T98G cell viability, profound cytotoxicity to the two cell lines was reported, thus highlighting the appropriateness of that system in the treatment of brain cancer [244]. Important findings originated from the work of Wang and his colleagues, where they proved that Verbascoside (VB) administration not only suppressed major cancer hallmarks, such as GBM cell propagation, infiltration, and migrative behavior as well as vessel-like tube formation, but also fostered miR-7-5p expression, which was found to be delivered to recipient GBM cells through exosomes and, consequently, exerted tumor-suppressive functions in vitro as well as diminished tumor development and metastasis in vivo [245]. The retardation of glioma cells’ propagation was achieved by DOX-encapsulated exosomes via microfluidics to increase the packaging ability of common anticancer drugs, such as DOX and PTX into exosomes. Exosomes from SF7761 glioma stem cells-like- and U251-glioma cells were segregated and identified by a plethora of different methods, and their study revealed the ability of DOX and PTX to be loaded in exosomes as well as that the transport of DOX to SF7761 glioma cells through their own exosomes was notably more effective and potent than with exosomes derived from U251 GMs, suggesting that using autologous exosomes may be more favorable for glioma drug delivery [246].

A novel self-assembly drug-delivery platform was synthesized after exosomes’ isolation from cell culture medium of the U87 cell line and, simultaneously, incubation with [Ag (GSH)]+ and DOX separately and combined. Studies with the aforementioned exosomes demonstrated the high-efficiency potential of silver nanoclusters (AgNCs) as smart cancer-targeting agents as compared to exosomes that lack AgNCs in parallel to high uptake and excessive ROS production with sustained DOX release in cancer cells and zero activity on normal cells [247].

An immunotherapy approach was adopted by Liu and his colleagues, where they designed a DC vaccine-based immunotherapy for GBM, which includes the co-delivery of tumor-derived exosomes with alpha-galactosylceramide with high effectiveness in controlling the release between immunoinhibitory and immunostimulatory cytokines. The same team showed that tumor-derived exosomes displayed a strong antitumor effect and when combined with invariant natural killer T (iNKT) cells, the immune tolerance was broken and a response against GBM cells was achieved [248].

### 4.6. Blood Exosomes

An attractive approach for high-yield large messenger RNA (mRNA) exosomes’ encapsulation was followed by Yang and his team. In their study, they introduced a method involving the release of blood-isolated exosomes carrying the desired mRNA, in this case PTEN mRNA. This was achieved by the stimulation of the donor cells with a focal and transient electric stimulus [249]. Indeed, the previously mentioned method generated up to 50-fold more exosomes and 10^3^-fold augmentation in exosomal mRNA transcripts, therefore underlining the improved yields compared to conventional exosomes production strategies. For instance, their results revealed that exosomes containing PTEN mRNA restored tumor-suppressive functions, increased the restraint of tumor expansion, and enhanced survival in mouse models of orthotopic PTEN-deficient glioma [249].

An effective nanoplatform, in which DOX and miR-21 inhibitor (miR-21i) were co-embedded into blood exosomes, was designed by Zhan and his colleagues [250]. Simultaneously, the magnetic moieties and endosomolytic peptides L17E were bound to the exosomes’ membrane through coupling between the ligand and the receptor and charge interactions, respectively, in order to generate exosomes with improved tumor accumulation and efficient delivery of cargo in tumor cells under an external magnetic field [248]. Firstly, it was shown that the engineered and labeled exosomes accelerated the endocytic uptake and enhanced their tumor accumulation ability [248]. In addition, the researchers found an improved and synergistic antitumor effect in mice injected with L17E-exosomes encapsulated DOX and miR-21i as compared to exosomes without the L17E peptide and embedded only either DOX or miR21i, thus emphasizing the potential of combined therapeutic effects of the agents used [250]. In another work, two STAT3 inhibitors, tanshinone IIA (TanIIA) and glycyrrhizic acid (GL), were selected to construct self-assembled TanIIA-GL nanomicelles (TGM) followed by encapsulation in serum exosomes and, simultaneous, the anchoring of CpG oligonucleotides, which are considered as agonists of TLR9 on the exosomes membrane to form immune exosomes loaded with TCM self-assembled nanomicelles (CpG-EXO/TGM) [251]. It was found that the aforementioned exosomes bound to free transferrin in serum with extended blood circulation time and maintain structure when crossing the BBB to directly target GBM cells [251]. In vivo results in orthotopic glioma mice model revealed that CpG-EXO/TGM significantly lengthened median survival, and when co-administered with TMZ, a synergistic curative effect was observed and a postoperative recurrence, an important challenge in GBM treatment, was inhibited [251]. The same combinatorial effects were observed in the work of Zhan and his colleagues, where blood exosomes were selected and loaded with cytosolic Phospholipase A2 (cPLA2) siRNA as well as with the antidiabetic drug metformin to target GBM energy metabolism, and their results revealed increased cellular uptake and strong antitumor effects in vitro. In vivo experiments demonstrated an inhibition of tumor growth and significant extension of mice survival time. Indeed, the simultaneous cPLA2 siRNA and metformin delivery held the most prominent inhibitory effect on GBM progression [252]. The effectiveness of drug delivery between the free and the exosome-encapsulated drug is discussed in Table 1.

### 4.7. Natural Brain Endothelia Cell-Derived Exosomes

The BBB, which protects the brain from pathogens and controls the passage of circulatory components, mostly consists of brain endothelial cells (BECs) [256]. The successful loading of siRNAs for the *VEGF* gene was demonstrated by Yang and his colleagues, where, firstly, they proved the knockdown of the aforementioned gene. As a consequence, an inhibition in VEGF protein expression was recorded and, secondly, they displayed the ability of brain endothelial cells (bEND.3)-derived exosomes to cross the BBB, effectively deliver their cargo and hinder the aggregation of xenotransplanted cancer cells in zebrafish [257]. Moreover, exosomes’ contribution to brain cancer treatment became clear through the work of Yang and his colleagues, where they evaluated the potential of exosomes isolated from brain endothelial bEND.3 cells to deliver DOX and PTX into the brain with high intracellular uptake and cytotoxicity of U87 cells in vitro as well as studied the increased BBB penetration efficiency in vivo in *Danio rerio* [255].

### 4.8. 293T Cell-Derived Exosomes

A very promising effort was conducted by Kim and his colleagues, who utilized the ligand of the GBM surface-overexpressed transferrin receptor, the T7 peptide, to ensure the selectivity and the targeted accumulation of the delivered exosomes [258]. Antisense miRNA oligonucleotides against miR-21 (AMO-21) were encapsulated into exosomes derived from the 293T cell line by electroporation, and the engineered T7-labeled exosomes were studied for their apoptosis-inducing ability [258]. In vitro experiments revealed an increased delivery efficiency to C6 GBM cells as compared to unmodified exosomes. The in vivo experiments proved a profound reduction in miR-21 expression, which is a phenomenon that induced the expression of programmed cell death protein 4 (*PDCD4*) and *PTEN*, resulting in the shrinkage of tumor sizes [258]. Furthermore, the exosomal transfer of the miR-21-sponge construct successfully downregulated miR-21 expression in glioma cell lines U87MG and C6. As a result, miR-21 negative regulation led to the upregulation of miR-21 target genes, such as *PDCD4* and reversion inducing cysteine rich protein with kazal motifs (*RECK)*. Consequently, a decreased rate of cell proliferation and an increased apoptotic rate were observed both in vitro and in vivo. In addition, the administration of exosomes loaded with a miR-21 sponge construct in a rat model of GBM revealed retention in tumor growth, inducement of tumor retardation as well as a notable decrease in the size of the tumors [259].

The contribution of exosomes isolated from 293T cells, conjugated with anti-CD22 monoclonal antibody fragments (CD22-F(ab’)_2_) and DOX-encapsulated, was studied in primary central nervous system lymphoma (PCNSL) with an increased apoptosis rate in vitro and extended life expectancy as well as enhanced antitumor activity in vivo [253]. The independent intranasal administration of curcumin-encapsulated exosomes or STAT3 inhibitor-encapsulated exosomes revealed a significant abortion in tumor growth and an increase in mice survival compared to the control-treated group [254].

### 4.9. Milk Exosomes

Exosomes derived from milk have also been successfully tested as natural NPs for effective siRNA delivery. siRNAs against specific genes including *VEGF*, *EGFR*, Protein kinase B (*AKT)*, and mitogen-activated protein kinase (*MAPK*), as well as Kirsten rat sarcoma virus (*KRAS*) were loaded in milk exosomes with electroporation. Results demonstrated the decrease in expression level from 2 to 10-fold in different cancers of the aforementioned genes, thus representing a novel therapeutic weapon against cancer [260].

Consequently, exosomes derived from different cell sources and involved in brain cancer treatment are listed in the table below (Table 2). This table aims to highlight the synergistic effect of the simultaneous delivery of both biomacromolecules and drugs as exosomal-encapsulated cargo applied in brain cancer therapy. The encapsulated cargo may be either biomacromolecules or drugs, while two cases were reported in which the isolated exosomes were delivered directly in vitro or in vivo without bearing any encapsulated cargo [208,234]. In many cases, only one type of cargo was loaded, and as a result, the other type of cargo is designated as not present (n.p.). The outcome is recorded only where studies have been conducted, but in certain cases, the in vitro or in vivo effect is not studied (n.s.).

## 5. Concluding Remarks

The exploitation of the advantageous properties of exosomes, such as their potential to function as targeted drug vehicles, may amplify the accumulation of therapeutic agents into brain tumors and avoid the distribution in physiological brain tissue, thus reflecting overall the improvement of therapeutic effectiveness. Recent scientific sources tend to employ various combinational attempts to achieve the simultaneous delivery of various therapeutic agents tailored to the patient’s needs, and this will be multiplied, given the emergence of a cascade of novel drugs available for use and the potential to administer cocktails of various drugs for personalized medicine. Taking the plethora of exosomal cargos that could be delivered by exosomes into consideration, it is deduced that the wide spectrum of applications in the diagnostic and therapeutic fields will arise. Moreover, a novel route will be expected by the further exploitation of the combination of the therapeutic and the imaging potential provided to exosomes as cargo to target brain tumors.

Exosomes originated from cancer cells are considered as critical factors contributing to the biogenesis of glioma and the advanced tumor progression. In addition to this, they constitute a contingent pool for fishing miRNAs biomarkers suitable for diagnosis and monitoring cancer development in patients suffered from gliomas. However, concerning the exosomes-involved treatment, it is important to take into account that neoplastic exosomes may perform the delivery of cargos preferentially to tumors in contrast to normal cells due to their homotypic features, thus opening an avenue for exploiting exosomes in various applications of drug-targeted administration. Among the several applications of exosomes, the principal ones lie in the clinical field including their use as cell-free therapeutic agents, drug delivery vehicles, biomarkers, cancer vaccines, and studies for exosome kinetics. The diagnosis-discussed methods and techniques, especially after the COVID-19 pandemic, are common procedures introduced in every diagnostic center equipped with a PCR thermocycler, while experimental assays are conventional and easy to cope with. Undoubtedly, the experience of working staff and an in-depth understanding of the utilized technique are requirements to minimize the occurring errors during the experimental procedure. Furthermore, other exosome sources, such as fruits, vegetables, and alternative types of milk, could potentially be investigated for the isolation of efficiently active exosomes. Moreover, unraveling the employment of different cargo molecules, in case their chemistry permits the encapsulation into exosomes, such as drugs, miRNAs with a tumor-suppressing nature, or anti-miRNAs acting as expression suppressors of oncogenic miRNAs, would also be of great interest. In order to plan an individualized therapy strategy and provide more accurate therapeutic assays, it is crucial to elucidate the underlying molecular mechanisms that induce oncogenesis and genomic/microsatellite instability in different brain cancer forms, e.g., GBM, with the aim to intervene in the suppression of the generated phenotype [261,262,263].

Engineered exosomes and, occasionally, those labeled from different cell sources have been successfully tested to deliver various types of cargo, such as drugs, proteins, and RNA molecules including mRNA, siRNA, and miRNA as well as circRNA, while ASOs have also been auspiciously encapsulated both in vitro and in vivo. In a number of instances, two different therapeutic agents were encapsulated into exosomes displaying better anticancer effect compared to one encapsulated cargo, thus pointing out the need for combinatorial targeting to simultaneously abort signaling pathways implicated in hallmarks that enhance tumor progression and resistance to conventional therapeutic protocols.

In the field of brain cancer treatment, almost all research teams recorded spectacular in vitro results regarding the suppression in proliferation, invasion, and migration as well as TMZ chemosensitivity restoration in glioma cell lines. In parallel, in some cases, the self-renewal capacity of GSCs was diminished, which is a very promising effect that could potentially eliminate the tumor. The in vivo experiments revealed not only an efficient BBB penetration and tumor-targeting ability owing to their characteristic properties but also significant tumor growth inhibition, attenuation of metastatic potential, increase in mice survival, as well as low toxicity to adjacent normal tissues. As a consequence, it could be a bright solution for a challenging type of cancer. However, there are still inquiries that have to be answered. For instance, a plethora of anticancer drugs used in clinical practice should be examined for their efficient exosome encapsulation and the possible anticancer effect both in vitro and in vivo. Moreover, the investigation of signaling biomolecules that enhance neoplasm growth as well as the elucidation of their biological role could amplify the choices of encapsulation, such as enzyme and miRNAs inhibitors, siRNAs, ASOs as well as mRNA transfer for restoration of tumor-suppressor proteins.

Several exosomes of human or vegetable origin are already employed in numerous clinical trials. However, the clinical implementation of exosomes confronts diverse queries and challenges. This scientific field is evolving rapidly, and the investigation of their subjacent mechanisms may reveal roles of exosomes that urgently claim further multidisciplinary cooperation to complete the knowledge about the aforementioned vesicles. Furthermore, the outcomes of clinical trials that are exosome grounded are required to comply with particular GMPs depending on the kind of the performed application and the studied disease. In more detail, a GMP-grade exosome preparation method should be conducted, including the type of cells, the cultivation system, and in detail the medium and environment of the culture generally.

Another crucial step of exosomal GMP production is the selection and setting up of characterization and identification methods, assuring the exosomes’ physical configuration and features for bioactivity function [264]. Secondly, another crucial limiting factor for exosomal clinical use constitutes the absence of a high-quality and homogenous specimen of exosomes batch to batch due to poor reproducibility. In addition, the European Medicines Agency (https://www.ema.europa.eu/en, accessed on 22 March 2023) has announced guidelines for the scientific world concerning the categorization of modern medicinal formulations for therapy.

Despite the plethora of advantages that exosomes exhibit, there are also some limitations in their application. The first restrictive factor in the clinical use of exosomes is the inability to achieve large-scale quantities given that the beneficial dose of exosomes reported touches approximately 10–100 μg protein of exosomes/mouse in previous research [176,177]. However, the yield of exosomes usually reaches no greater than 1 μg protein of exosomes originating from 1 mL of the extracellular culture medium [265,266].

Although the obstacle in the production of high quantities of exosomes seems obvious, the in-progress clinical trials (6 clinical trials were deposited in ClinicalTrials.gov resource (https://clinicaltrials.gov/ct2/home, accessed on 23 March 2023), concerning the employment of exosomes for delivering therapeutic agents, resolved or rebutted this issue. However, it will be challenging enough to examine the therapeutic potential of autologous or non-autologous exosomes in treating brain cancer through studies in human brain organoids and, subsequently, clinical trials. As a result, there is an urgent need to plan, develop, organize, and conduct clinical trials for validating the curative effects of exosome-based therapies in brain malignancies. Even if exosomes were beautified enough in clinical trials, doubts have emerged, since recent evidence showed that three clinical trials were based on the modification of MSCs to overexpress exosomes. Nevertheless, none of these trials have been finished, and outcomes have not been published yet.

Based on the various exosome isolation methods, the greater issues that arise are the purity of exosomes reflecting other alterations of their physicochemical properties. Another element affecting the functionality of exosomes is the standardization of storage conditions. Moreover, a crucial point is the amplification of the therapeutic capacity/effectiveness of exosomes. An overexpression or enrichment of the therapeutic biomolecules may be attempted to overcome this problem of the low efficiency of exosomes, thus leading to an enhanced therapeutic potential. Last but not least, it should be mentioned that the delivery potential of exosomes in the area of the desired target tissue or cell constitutes a crucial issue, given the fact that the biological outcome of exosomes results from their internalization and their biodistribution into the target cells. Gaining insight into these topics is obligatory for clinically therapeutic applications.

Exosomes constitute an inspired natural structure mimicking a Trojan horse described in Greek mythology, which in fact was a construction that provided a hiding place for the most important ancient Greek heroes. In the case of exosomes, these particles are capable of transporting and protecting all the aforementioned precious cargos.

The investigation of the ensemble of the EVs named the “vesiculome” in patients suffering from brain tumors might possibly enrich science with unique insights such as the elucidation of oncogenic-triggered mutations and medication-resistance-correlated mutations, e.g., EGFR mutations, the unraveling of oncogenic pathways, the classification of disorder subgroups and molecular signatures. Similarly, gaining insight into the molecular profile of the tumor for the appropriate therapeutic personalized protocol administration could assist the encapsulation potential of the ideal drug candidate in exosomes to ensure the most potent anticancer effect and ideal cell or tissue targeting.

## Figures and Tables

**Table 1:** Comparison of drug delivery effectiveness between the free and the exosome-encapsulated drug.

Drug	Chemical Structure	Free	Exosome Encapsulated	Reference
5-FC	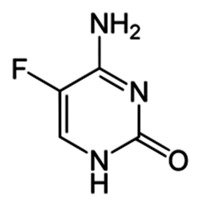	Non-active	Tumor growth suppression of C6 GBM cells	[199]
TMZ	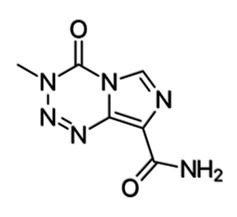	Non-active	Inhibition of the TMZ-resistant GBM growth in cell lines	[209,235]
Atorvastatin	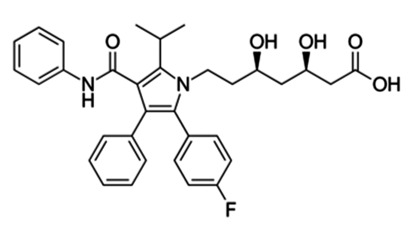	Reduced tumor-targeting efficiency	Tumor growth inhibition with reduced cell propagation, migration and VEGF secretion	[211,212]
BQR	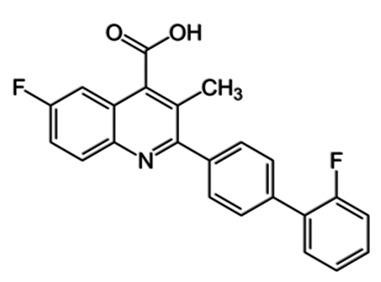	Limited BBB penetration	Improved ferroptosis, BBB penetration and tumor-targeting ability	[213]
DOX	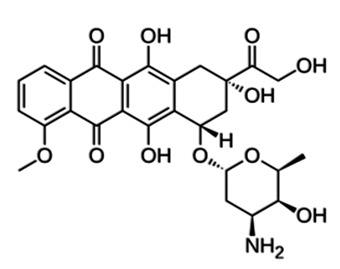	Limited BBB penetration	High apoptotic effect and tumor growth suppression	[216,231,239,247,250,253]
PTX	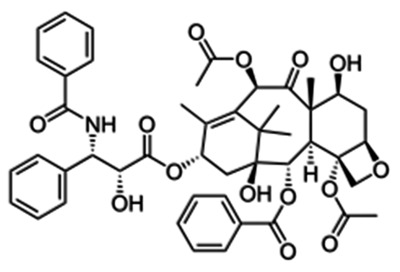	Limited BBB penetration	High cytotoxicity and improvement of therapeutic effects through optimized targeting	[219,244]
Cur	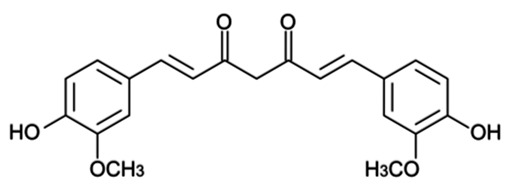	Limited BBB penetration and tumor-targeting ability as well as poor stability in liquid environment	Increased imaging and therapeutic effects with reduced side effects and augmented survival in mice models	[232,254]
ICG	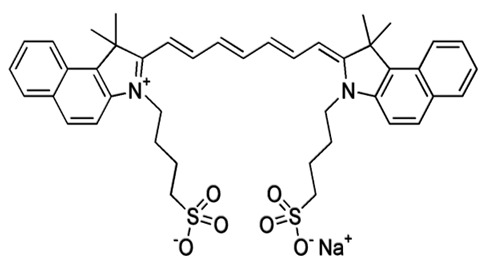	Limited BBB penetration, tumor-targeting ability and poor stability in aqueous solution	Relief of the intracellular hypoxia microenvironment, BBB penetration, tumor accumulation and potent anticancer effect	[233,240]
Panobinostat	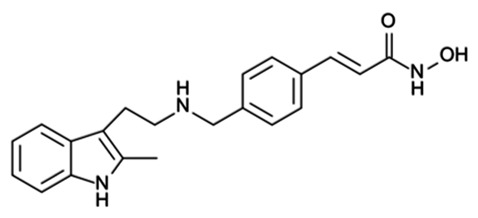	Limited BBB penetration and poor water solubility	Inhibition of DIPG cell proliferation	[236]
HCQ	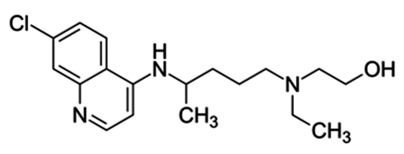	Limited BBB penetration and non-selective distribution of the drug in vivo	Strong antitumor activity	[217]
TMZ & DHT	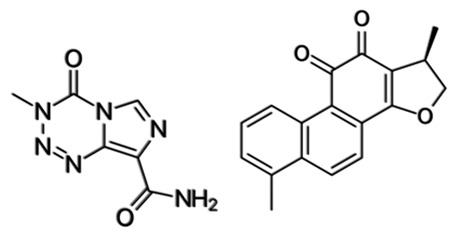	Non-active/TMZ-resistance inversion and apoptosis induction	Good antitumor efficacy and targeting of the tumor site of TMZ-resistant cells and mice models	[241]
Selumetinib	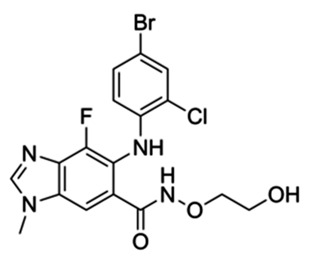	Limited BBB penetration	Strong antitumor effect with zero toxicity in vivo	[243]
VB	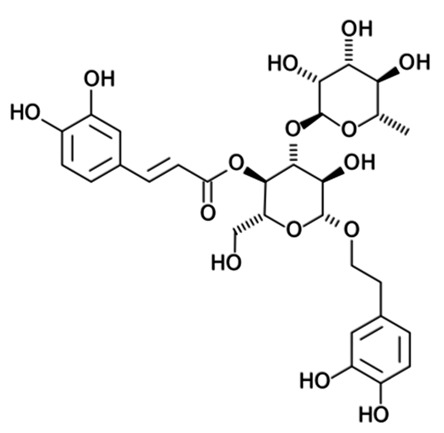	Strong anticancer effect in vitro and in vivo	Not studied	[245]
DOX & PTX	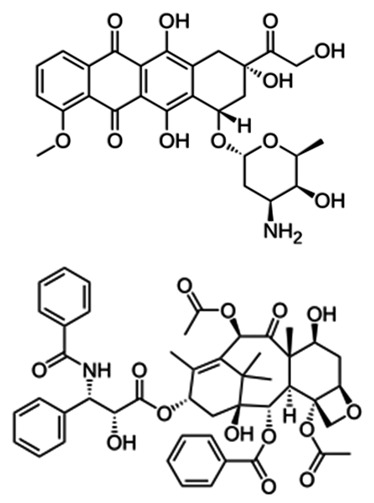	Limited BBB penetration	Autologous exosomes represent the most potent weapon for glioma drug targeting with increased cytotoxicity and BBB penetration	[246,255]
TanIIA and GL	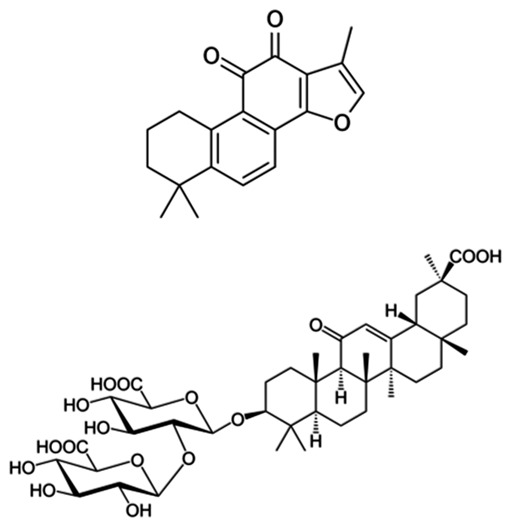	Poor solubility in water and quick metabolism/Self-assembly with fat-soluble ingredients for nanomicelle formation	Great BBB penetration, apoptosis inducement and when co-administered with TMZ an enhanced synergistic effect as well as an inhibition of postoperative recurrence was observed	[251]
Metformin	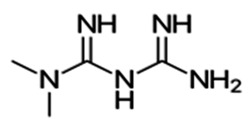	Limited BBB penetration, poor stability an inefficient cell uptake	Increased cell uptake, suppression of tumor growth and significant extension of mice survival time	[252]

**Table 2:** Exosomes derived from different cell sources that are involved in brain cancer treatment.

Cell Source	Encapsulated Cargo		Outcome In Vitro	Outcome In Vivo	Reference
	Macromolecule	Drug			
Mesenchymal stem cell-derived exosomes	yCD::UPRT	5-FC	Tumor growth inhibition of C6 GBM cells		[199]
	miR-124 and miR-145	n.p.	Reduced migration rate of tumor cells and diminished the regeneration capacity of GSCs	U87-derived xenograft penetration and downregulation of miR-124 target genes, such as CDK6	[200]
	Anti-miR-9	n.p.	Chemosensitivity, increased cell death as well as elevated caspase activity	n.s.	[201]
	miR-512-5p	n.p.	Cell propagation inhibition and cell cycle arrest induction	n.s.	[202]
	miR-199a	n.p.	Suppression in proliferation, invasion and migration as well as TMZ chemosensitivity induction	Repression of tumor growth	[203]
	miR-29a-3p	n.p.	Migration and vasculogenic mimicry formation attenuation		[204]
	miR-133b	n.p.	Suppression in proliferation, invasion and migration	n.s.	[205]
	miR-584	n.p.	Decreased proliferation and invasion rate of glioma cells	Attenuation of malignant gliomas development	[206]
	miR-146b	n.p.	n.s.	Glioma xenograft growth inhibition	[207]
	n.p.	n.p.	Apoptosis induction in C6 cells	n.s.	[208]
	STAT3-siRNA	TMZ	Inhibition of TMZ-resistant GBM growth		[209]
	miR-124a	n.p.	Significant reduction in viability and clonogenicity of GSCs	Tumor growth retention and increased survival	[210]
	n.p.	Atorvastatin	Reduced cell propagation, migration and VEGF secretion as well as increased apoptotic activity	n.s.	[211]
	n.p.	Atorvastatin	Tumor growth suppression in a 3D GBM model	n.s.	[212]
	GPX4-siRNA	BQR	Enhanced ferroptosis in GBM cell lines	BBB penetration and tumor-targeting ability	[213]
	miR-375	n.p.	Inhibition in cell propagation, migration and infiltration as well as apoptosis induction	Repression of xenograft tumor in nude mice	[214]
	lncRNA PTENP1	n.p.	Suppression of malignant phenotype of U87 cells	n.s.	[215]
	n.p.	DOX	Increased cytotoxicity against cancer cells	n.s.	[216]
	Cytosine deaminase and miR-34a	n.p.	Increased apoptosis	n.s.	[223]
	FGFR3-TACC3 siRNA	n.p.	Decreased cell viability	Improved survival in glioma-bearing mice without toxicity to adjacent normal tissues and affecting the wild-type FGFR3 or TACC3	[225]
Embryonic stem cells-derived exosomes	n.p.	PTX	Improvement in the therapeutic effects of PTX through optimized targeting	n.s.	[219]
Neural stem cells-derived exosomes	STAT3 ASOs	n.p.	Stimulation of the immunogenic activity of human dendritic cells or mouse macrophages	Inhibit growth of GL261 tumors	[228]
	miR-124-3p	n.p.	Glioma cell proliferation, migration, and invasion as well as FLOT2 expression suppression	Tumor growth inhibition in a mouse tumor xenograft model	[229]
Macrophage-derived exosomes	miR-21	DOX	High apoptotic effect in glioma cells	n.s.	[231]
	n.p.	Curcumin	Targeted imaging and curative effects with reduced side effects		[232]
	Catalase	ICG	Relief of the intracellular hypoxia microenvironment	BBB penetration, brain accumulation ability and strong anticancer effect	[233]
	n.p.	n.p.	n.s.	100% Mice survival ratio	[234]
	O6-benzylguanine (BG)	TMZ	Proliferation inhibition of U87MG and GSCs	n.s.	[235]
	PPM1D-siRNA	Panobinostat	DIPG cell proliferation inhibition	n.s.	[236]
	circBTG2	n.p.	Inhibition in cell proliferation and invasion	n.s.	[237]
	miR-15a and miR-92a	n.p.	Inhibition of malignant behavior of glioma cells	n.s.	[238]
	n.p.	DOX	Tumor growth suppression		[239]
Glioma cells-derived exosomes	n.p.	HCQ	High antitumor potency with visible light illumination		[217]
	n.p.	ICG	n.s.	Abolishment of tumor growth	[240]
	n.p.	TMZ and DHT	Good antitumor efficacy and targeting of the tumor site of TMZ-resistant cells and mice models		[241]
	miR-151a	n.p.	chemosensitivity recovery to TMZ		[242]
	n.p.	Selumetinib	Strong antitumor effect	Strong antitumor effect with zero toxicity	[243]
	n.p.	PTX	High cytotoxicity to U87 and T98G cell lines	n.s.	[244]
	miR-7-5p	VB	Inhibition of cell proliferation, invasion, and migration as well as vessel-like tube formation	Diminish tumor formation and metastasis	[245]
	n.p.	DOX and PTX	Autologous exosomes represent the most potent weapon for glioma drug targeting	n.s.	[246]
	[Ag (GSH)]+	DOX	High glioma cells uptake and constant DOX release in cancerous cells	n.s.	[247]
	alpha-Galactosylceramide	n.p.	n.s.	Stimulation of tumor-specific cytotoxic T lymphocytes proliferation, synergistically breaking the immune tolerance and improving the immunosuppressive environment	[248]
Blood exosomes	n.p.	DOX	Neoplastic growth suppression		[239]
	PTEN mRNA	n.p.	n.s.	Restoration of tumor suppression, enhancement of tumor growth inhibition, and improvement of survival in an orthotopic PTEN-deficient glioma mouse	[249]
	n.p.	DOX and miR-21i	Acceleration of the endocytic uptake and enhanced tumor accumulation ability	Improved and synergistic antitumor effect	[250]
	n.p.	TanIIA and GL	Great BBB penetration and apoptosis inducement in GL261 cells	Extension of mice median survival and when co-administered with TMZ, a synergistic therapeutic effect was recorded as well as inhibition of postoperative recurrence	[251]
	cPLA2-siRNA	Metformin	Increased cellular uptake and strong antitumor effect	Inhibition of tumor growth and significant extension of mice survival time	[252]
Natural brain endothelia cell-derived exosomes	VEGF-siRNA	n.p.	BBB penetration efficiency and increased cellular uptake	Strong BBB penetration and aggregation abortion of xenotransplanted cancer cells	[257]
	n.p.	DOX & PTX	High cytotoxicity and intracellular uptake to U87 cell line	Efficient BBB penetration	[255]
293T cell-derived exosomes	AMO-21	n.p.	Increased delivery efficiency to C6 GBM cells	Expression induction of PDCD4 and PTEN as well as tumor shrinkage	[258]
	miR-21-sponge construct	n.p.	Cell proliferation reduction and increased apoptosis	Tumor growth retention and tumor retardation inducement as well as reduction in tumors volume	[259]
	n.p.	DOX	Increased apoptosis	Life expectancy extension and antitumor activity enhancement	[253]
	n.p.	Cur/ STAT3-inhibitor	n.s.	Abortion in tumor growth and mice survival increase	[254]
Milk exosomes	siRNA against a plethora of oncogenes	n.p.	Knockdown of oncogenes and reduced cell proliferation	n.s.	[260]

n.p.: not present; n.s.: not studied.

## Data Availability

Not applicable.

## Abbreviations

5-FC	5-Fluorocytosine
5-FU	5-Fluorouracil
14-3-3E	14-3-3 protein epsilon
AGAP2	ArfGAP with GTPase Domain, Ankyrin Repeat And PH Domain 2
AgNCs	Silver nanoparticles
Agos	Argonautes
AKT	Protein kinase B
ANXA1	Annexin A1
ANXA2	Annexin A2
APC	Adenomatous polyposis coli
APNG	Alkylpurine-DNA-N-glycosylase
ARF6	ADP Ribosylation Factor 6
ASOs	Antisense Oligonucleotides
ATG12	Autophagy Related 12
B7-H1	B7-Homolog 1
BAF-TiN	Biotinylated Antibody-Functionalized Titanium Nitride
BBB	Blood-brain-barrier
BECs	Brain Endothelial Cells
BG	Benzylguanine
BM	Brain metastases
BMSC	Bone mesenchymal stem cells
BMSCExo	Bone marrow mesenchymal stem cells-derived exosomes
BNCT	Boron Neutron Caption Therapy
BQR	Brequinar
BPA	Boron Phenylalanine
BTG2	B-cell translocation gene 2
CA9	Carbonic Anhydrase 9
CCND1	Cyclin D1
CD	Cluster of Differentiation
CD117	KIT proto-oncogene receptor tyrosine kinase
CDA	Cytosine Deaminase
CDK6	Cyclin-dependent kinase 6
circRNA	circular RNA
COF1	Cofillin
COP I	Coat protein I
CpG	5′—C—phosphate—G—3′
cPLA2	cytosolic Phospholipase A2
CSF	Cerebrospinal fluid
CT	Computed Tomography
Cur	Curcumin
CYP2J2	Cytochrome P450 2J2 isoform
DCs	Dendritic Cells
DHODH	Dihydroorotate Dehydrogenase
DHT	Dihydrotanshinone
DIPG	Diffuse Intrinsic Pontine Glioma
DNA	Deoxyribonucleic acid
DNM3	Dynamin 3
DOX	Doxorubicin
dsDNA	Double-stranded DNA
ECM	Extracellular Matrix
EGFR	Epidermic Growth Factor Receptor
EGFRvIII	Epidermal Growth Factor Receptor variant III
ENOA	Alpha-enolase
ESCRT	Endosomal Sorting Complex Required for Transport
ESCs	Embryonic Stem Cells
EVs	Extracellular vehicles
EZH2	Enhancer of Zeste 2 Polycomb Repressive Complex 2 Subunit
FasL	Fas Ligand
FBXO11	F-Box Protein 11
FGFR	Fibroblast Growth Factor Receptor
FLOT2	Flotillin 2
FOXA2	Forkhead box protein A2
FUS	Focused Ultrasound
G3P	Glyceraldehyde-3-phosphate dehydrogenase
GATA2	GATA-binding factor 2
GBM	Glioblastoma
GL	Glycyrrhizic acid
GLI1	GLI family zinc finger 1
GMP	Good manufacturing practice
GPX4	Glutatheione peroxidase 4
GSCs	Glioblastoma Stem Cells
hEnMSCs	Human Endometrial Mesenchymal Stem Cells
HCQ	Hydroxychloroquine
HGA	High-grade astrocytoma
HGG	High-Grade Glioma
HIF-1a	Hypoxia-inducible factor-1a
HIFs	Hypoxia-inducible factors
HLA-G	Human leukocyte antigen-G
HMOX1	Heme oxygenase 1
hMSCs	Human Mesenchymal Stromal Cells
HSP90B	Heat-shock protein 90B
HSPs	Heat-shock proteins
HSSP	Heme oxygenase-1 specific short peptide
hUC-MSCs	Human Umbilical Cord Mesenchymal Stem Cells
HUVEC	Human Umbilical Vein Endothelial Cells
ICAM-1	Intercellular Adhesion Molecule-1
ICG	Indocyanine green
ICOSL	Inducible costimulator ligand
ICP-MS	Inductively coupled plasma mass spectometry
IDH1	Isocitrate dehydrogenase-1
IFN-g	Interferon-gamma
IL-10	Interleukin-10
IL-12	Interleukin-12
IL-3	Interleukin-3
iNKT	Invariant Natural Killer T
ISEV	International Society for Extracellular Vesicles
JAG1	Jagged Canonical Notch Ligand 1
KRAS	Kirsten rat sarcoma virus
LAMP-1	Lysosomal-Associated Membrane Protein-1
LAMP-2b	Lysosomal-Associated Membrane Protein-2b
LGG	Low-grade glioma
LLGL2	LLGL Scribble Cell Polarity Complex Component 2
lncRNA	Long non-coding RNA
MAPK	Mitogen-activated protein kinase
MaSC	Marrow stromal cells
MGMT	O(6)-methylguanine DNA methyltransferase
MHC-I	Major histocompatibility complex-I
MHC-II	Major histocompatibility complex-II
miRNA	MicroRNA
MRI	Magnetic Resonance Imaging
mRNA	Messenger RNA
MSCs	Mesenchymal stem cells
mTOR	Mammalian target of Rapamycin
ΜVs	Microvesicles
MVBs	Multivesicular bodies
MVP	Major Vault Protein
NF-κB	Nuclear Factor κΒ
NHEJ	Non-homologous end joining
NIR	Near-infrared radiation
NPM	Nucleophosmin
NPs	Nanoparticles
NSCs	Neural Stem Cells
PCNSL	Primary Central Nervous Lymphoma
PCR	Polymerase chain reaction
PD-1	Programmed Death-1
PDA	Polydopamine
PDCD4	Programmed cell death protein 4
PD-L1	Programmed Death-Ligand 1
PI3K	Phosphoinositide 3-kinase
PKM	Pyruvate Kinase M1/2
PPM1D	Protein Phosphatase 1D
PRDX1	Peroxiredoxin-1
PRKDC	Protein Kinase, DNA-Activated, Catalytic Subunit
PTEN	Phosphatase and tensin homolog
PTENP1	Phosphatase and tensin homolog pseudogene 1
PTX	Paclitaxel
Rab	Ras-associated binding
RAB13	Ras-related protein 13
Rac1	Ras-related C3 botulinum toxin substrate 1
RAP1B	Ras-associated protein 1B
Rb	Retinoblastoma protein
RECK	Reversion Inducing Cysteine Rich Protein With Kazal Motifs
R-EXO	Reassembly-Exosomes
RNA	Ribonucleic acid
RNU6-1	RNA, U6 Small Nuclear 1
ROS	Reactive Oxygen Species
rRNA	Ribosomal RNA
RVG-15	Rabies viral glycoprotein
SCI	Spinal Cord Injury
SEC	Size-exclusion chromatography
siRNA	Small-interfering RNA
SLC31A1	Solute Carrier Family 31 (Copper Transporter), Member 1
sncRNA	Small non-coding RNA
SPIONs	Superparamagnetic Iron Oxide Nanoparticles
ST14	ST14 Transmembrane Serine Protease Matriptase
STAT3	Signal transducer and activator of transcription 3
TACC	Transforming Acidic Coiled-Coil Containing Protein 3
TanIIA	Tanshinone IIA
TdEX	Tumor-derived Exosomes
TERA	Transitional endoplasmic reticulum ATPase
TFF	Tangential Flow Filtration
TIMP3	Tissue inhibitor of metalloproteinase 3
TLR-9	Toll-like receptor 9
TMZ	Temozolomide
TPIS	Triose phosphate isomerase
TPM1	Tropomyosin 1
TPM4	Tropomyosin 4
tRNA	Transfer RNA
TSG101	Tumor susceptibility gene 101
UC	Ultracentifiguration
UTR	Untranslated region
VB	Verbascoside
VCP	Vasolin-containing protein
VEGF	Vascular Endothelial Growth Factor
VIME	Vimentin
WHO	World Health Organization
yCD::UPRT	Yeast cytosine deaminase::uracilphosphoribosyl transferase
ZEB1	Zinc Finger E-Box Binding Homeobox 1
ZnS	Zinc sulfide
